# Community-based pediatric palliative care for health related quality of life, hospital utilization and costs lessons learned from a pilot study

**DOI:** 10.1186/s12904-016-0138-z

**Published:** 2016-08-03

**Authors:** Jeffrey Goldhagen, Mark Fafard, Kelly Komatz, Terry Eason, William C. Livingood

**Affiliations:** 1 Division of Community and Societal Pediatrics, Department of Pediatrics, UF College of Medicine – Jacksonville, 841 Prudential Drive, Suite 1330 m, Jacksonville, FL 32207 USA; 2 Baptist Health Research Institute, Baptist Health System, 836 Prudential Drive, Pavilion 6th Floor, Jacksonville, FL 32207 USA; 3 Community PedsCare, Community Hospice of Northeast Florida, 4266 Sunbeam Rd., Jacksonville, FL 32257 USA; 4 Center for Health Equity and Quality Research, UF College of Medicine-Jacksonville, 580 W. 8th St., Tower II, Room 6015, Jacksonville, FL 32209 USA

**Keywords:** Pediatric palliative care, Chronic disease, Cost-effectiveness, Hospital utilization, Health related quality of life, Pilot study

## Abstract

**Background:**

Children with chronic complex-medical conditions comprise a small minority of children who require substantial healthcare with major implications for hospital utilization and costs in pediatrics. Community-Based Pediatric Palliative Care (CBPPC) provides a holistic approach to patient care that can improve their quality of life and lead to reduced costs of hospital care. This study's purpose was to analyze and report unpublished evaluation study results from 2007 that demonstrate the potential for CBPPC on Health Related Quality of Life (HRQoL) and hospital utilization and costs in light of the increasing national focus on the care of children with complex-medical conditions, including the Affordable Care Act's emphasis on patient-centered outcomes.

**Methods:**

A multi-method research design used primary data collected from caregivers to determine the Program's potential impact on HRQoL, and administrative data to assess the Program's potential impact on hospital utilization and costs. Caregivers (*n*=53) of children enrolled in the Northeast Florida CBPPC program (Community PedsCare) through the years 2002-2007 were recruited for the Health Related Quality of Life (HRQoL) study. Children (*n*=48) enrolled in the Program through years 2000-2006 were included in the utilization and cost study.

**Results:**

HRQoL was generally high, and hospital charges per child declined by $1203 for total hospital services (*p*=.34) and $1047 for diagnostic charges per quarter (*p*=0.13). Hospital length of stay decreased from 2.92 days per quarter to 1.22 days per quarter (*p*<.05).

**Conclusion:**

The decrease in hospital utilization and costs and the high HRQoL results indicate that CBPPC has the potential to influence important outcomes for the quality of care available for children with complex-medical conditions and their caregivers.

**Electronic supplementary material:**

The online version of this article (doi:10.1186/s12904-016-0138-z) contains supplementary material, which is available to authorized users.

## Background

Medical care increasingly extends the lifespan of chronically ill children without curing their underlying diseases or conditions [1, 2]. The high probability that children with chronic medical conditions will endure life necessitating extensive medical care, painful procedures and surgical interventions has created a growing consensus that quality of life for these children and families should be a priority [3–7]. Although children with chronic complex medical conditions comprise less than 5 % of the overall child population, their impact on the healthcare system is substantial [8]. In most European countries, these children’s illnesses are characterized by periods of frequent and prolonged hospitalizations [9]. In the United States, these children constitute 10 % of admissions to children’s hospitals [10] and 25 % of all hospital bed days; [3] and account for approximately 40 % of total Medicaid spending on children, 15–33 % of pediatric health care costs [11, 12] and 80 % of pediatric inpatient costs [11]. Nearly 50 % are dependent upon technology—10 % require feeding tubes, 7 % central venous catheters and 1 % tracheostomies [13]. Approximately 12 % are dependent on five or more medications [13, 14]. Re-admission rates for these patients have been shown to vary from 17 % to 66 % [8, 11]. On average, care for these children requires 13 different physicians from six distinct medical sub-specialties per child [15, 16]. And sadly, they represent 43 % of childhood deaths [10].

In response to the challenges of caring for these children, pediatric palliative care has emerged as an Accreditation Council for Graduate Medical Education (ACGME) boarded sub-specialty (Pediatric Hospice and Palliative Medicine) that evolved from a singular focus on end-of-life care to one emphasizing relief of suffering and quality of life [3, 6, 7, 17, 18]. Pediatric palliative care provides a continuum of interdisciplinary medical and psychosocial support services to children and families that seeks to: a) manage symptoms and relieve physical, emotional, psychological and social distress produced by medical conditions; [19, 20] b) help children with chronic and debilitating conditions, and their siblings and extended families, live as normally as possible and improve their quality of life [21], c) provide timely and accurate information to support children, families and caregivers in decision-making [22], d) empower children and parents to actively participate in decisions related to their care [23], and e) prepare the child and family for death by supporting them and their caregivers through the final months of terminal medical conditions and bereavement [17, 24].

As an important component of the palliative care continuum, community-based pediatric palliative care (CBPPC) enhances and extends the medical home model to engage children and families in their homes, schools and communities to ensure there is a holistic continuum of palliative care across primary care, hospital and community settings. CBPPC begins at the time of diagnosis (including prenatally) and continues through the life course of the child—including, if the child survives, transition into adulthood [25]. CBPPC home and community-based services that focus on health literacy and communication, medical decision-making and psychosocial support and case management have the potential to improve the health related quality of life (HRQoL) of children and families and reduce healthcare utilization and costs [11, 25–27].

In light of the relevance of CBPPC to the care of children with chronic complex medical conditions, it has become increasingly important to quantify its impact on quality and cost of care. With respect to quality, measures of health-related quality of life (HRQoL) provide insight into the granular effect of CBPPC on people’s lives. HRQoL goes beyond direct measures of population health, life expectancy and causes of death, and focuses on the impact health status has on quality of life. Measuring HRQoL over time using survey instruments: a) offers insight into the needs of the child, b) identifies the priority and extent of services that are required by the family to improve their quality of life and c) provides metrics to assess the impact of CBPPC interventions. Health care costs are more complex and challenging to evaluate over time, as actual costs and reimbursement data are difficult to obtain and analyze. In addition, despite the potential effectiveness of interventions, the child’s health status may continue to decline over time resulting in an increase in the utilization of health care services and associated costs. Despite these challenges, it is more important than ever to identify and measure the metrics of quality and cost of care as they relate to the impact of CBPPC on the health and well-being of children with chronic complex medical conditions.

In 2001, Community PedsCare was established by Community Hospice of Northeast Florida as a CBPPC program to provide comprehensive and compassionate palliative and end-of-life care to children with life-threatening, complex chronic conditions and their families. The program is designed to relieve suffering, provide comfort and improve overall quality of life. It provides community-based medical, nursing, social work, child life, spiritual and volunteer care in collaboration with Wolfson Children’s Hospital, Nemours Children’s Clinic, the University of Florida and multiple other community agencies. Services include pain and symptom management; medical consultation; mental health, psychosocial and spiritual support and counseling; family respite; assistance with financial issues and resource development; case management and care coordination; and bereavement and grief support. Special attention is also paid to the needs of the siblings. The program serves children under the age of 21, regardless of their financial status or insurance coverage.

### Purpose

In light of the rapid advances in the development and high global demands of pediatric palliative care [9], and the Affordable Care Act’s increased emphasis on patient centered outcomes, the purpose of this manuscript is to analyze and report unpublished evaluation study results from 2007 that demonstrate the potential for positive impact of Community PedsCare’s community-based palliative care program on HRQoL and hospital utilization (length of stay) and cost (facility, drugs, procedures, equipment, etc.). In addition to the outcome data, this evaluation retrospectively serves as a pilot [28] study for data collection methods, cost analysis models and HRQoL assessment tools specifically designed for palliative care research. This study attempts to provide preliminary answers to two evaluation research questions concerning community-based palliative care for children with life limiting and life threatening conditions. *1) What is the impact of community-based pediatric palliative care on quality of life? 2) How does community-based pediatric palliative care impact hospital utilization and related costs?* Publication of this study’s results provides an opportunity to contribute to the developing evidence base that supports CBPPC contribution to HRQoL and health care cost reduction. Our expectation is that this pilot project will inform and serve as guidance for future research and policy development.

## Methods

A multi-method research design was employed to answer the research questions. Primary data was collected from caregivers to determine the Program’s potential impact on health related quality of life. Secondary data was collected for the analyses to assess the impact of the Program on hospital utilization and costs. It was hypothesized that for children enrolled in Community PedsCare, quality of life would improve for clients and care givers and hospital utilization and costs would decrease.

Selection criteria included: Clients (0–18) who were enrolled in Community PedsCare (admissions range from 2002 to 2007) at the time of the study who had documented hospital admissions during the 2 years prior to and the first two quarters after enrolment in the program between 2002–2006. Criteria for admission to PedsCare were broadly defined to include all chronic life-limiting conditions (with new diagnosis, change in status, complex situation) including children already enrolled in hospice.

Parents/caregivers were invited to participate in the HRQoL study and consented through mailed letter and telephone invitations. The interviewer received consents from participants and recorded results through a paper survey. To ensure confidentiality, no names were linked to results and participants were identified by arbitrary unique codes during the analysis. Data was entered in a pass code protected secured Access database.

**Fig. 1:**
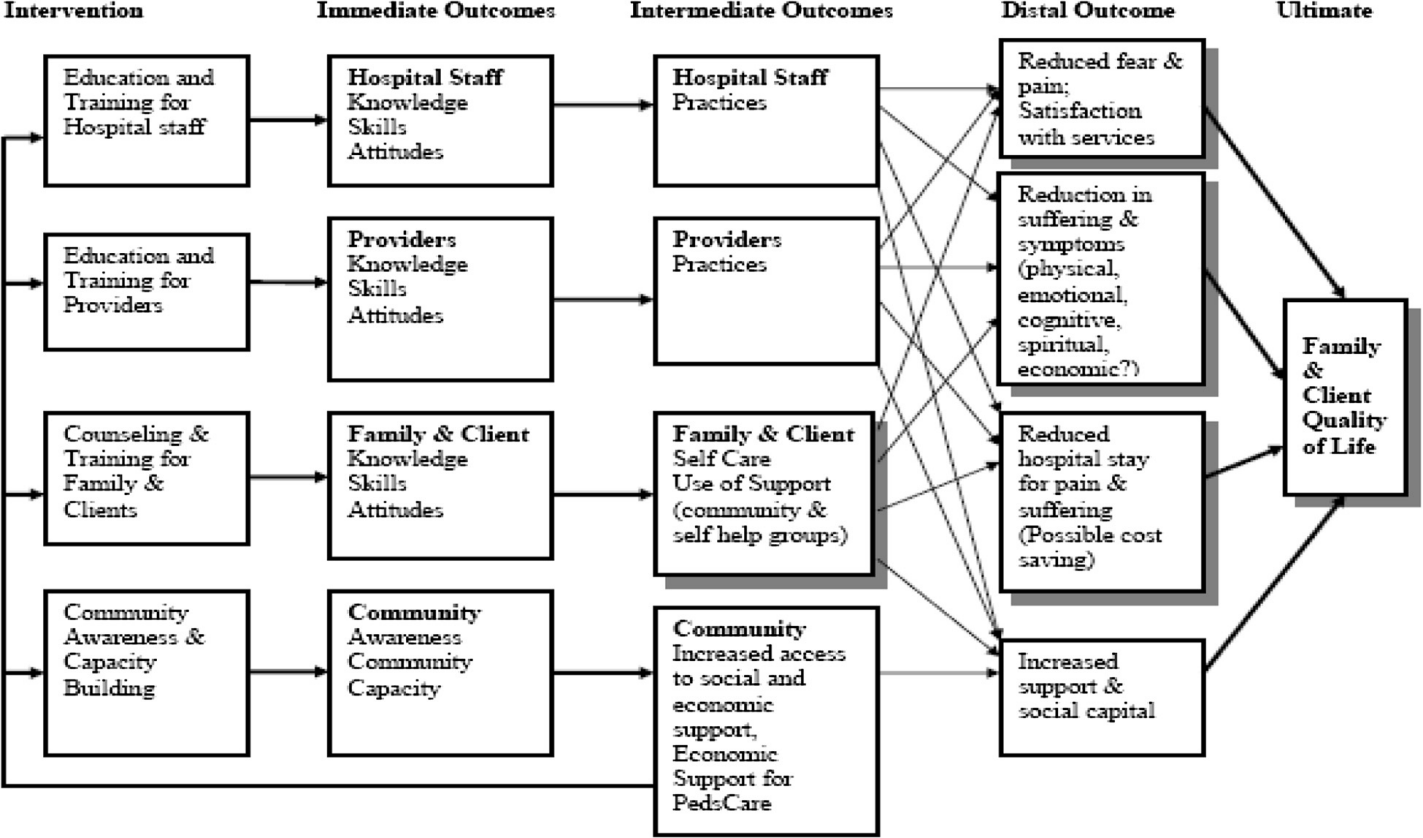
Evaluation logic model pediatric palliative care

In addition to the feasibility of obtaining related data, the outcomes that were selected for the HRQoL instrument, as well as the framework for the evaluation of this pilot study were based on the logic model below (see Fig. 1).

### Health related quality of life instrument development

The HRQoL instrument was developed by Community PedsCare and intended to pilot the collection of primary data from patients and caregivers focused on health related quality of life issues that could potentially be impacted by Community PedsCare palliative care services. Development of the instrument involved the following steps.

1. The scientific literature on HRQoL was reviewed and constructs from the literature defined. Although pre-existing validated HRQoL tools exist in Palliative Care, it was the intention to pilot a tool designed for Community Based Pediatric Palliative Care, particularly for Community PedsCare.
2. In-depth interviews of Community PedsCare staff and primary healthcare providers were conducted to obtain their perceptions of current and priority Community PedsCare services and desired Program outcomes.
3. HRQoL constructs/themes were abstracted from in-person interviews of Community PedsCare staff and primary care physicians.
4. Constructs from the literature were synthesized with the constructs that emerged from the local interviews.
5. The HRQoL instrument was refined and validated through a local expert panel review.

The scientific literature on HRQoL for pediatrics is extensive including articles on HRQoL developed and/or tested for specific conditions [29, 30], more generic HRQoLs for children in general [30–32], and pediatric HRQoL adapted from adult instruments [33]. These instruments tend to have similar constructs such as physical, social, emotional and overall functioning and tend to rely on child self-report and parental proxy report. Notably, these instruments tend to focus on the child’s HRQoL. The in-depth interviews that were used to adapt HRQoL concepts to Palliative Care revealed a holistic or social ecological [34] approach to care that viewed the whole family as receiving supportive care. Consequently, the instrument developed for this population utilized concepts of HRQoL found in over 30 years of literature, but in this case, specifically adapted to the unique focus of the palliative care program, the parents/caregivers [29–35]. The resulting HRQoL survey instrument was comprised of two parts. Part I measured the average number of days within the past 30 days that the respondents experienced conditions categorized under three constructs: general emotional health, respite care and activity limitation. The part had 7 items organized under 3 constructs. Part II was constructed to assess performance of the health care system and perceptions of parents, guardians and children related to their psychosocial and emotional health on an ordinal scale from 1 to 5. A total of seventeen (17) items were included under 5 constructs: decision-making, social support, interaction and communication, access to resources and child health.

### Data collection and analysis

Family/caregivers were invited to participate in the HRQoL study and consented through mailed letter and phone invitations. A contracted evaluation interviewer received verbal consents from participants and recorded results through a paper survey. To ensure confidentiality, no names were linked to results and participants were identified by arbitrary unique codes. Surveys were administered in the later part of 2007. HRQoL data was collected on the following Community PedsCare services provided to children and families: pain and symptom management; medical consultation; mental health, psychosocial and spiritual support and counseling; family respite; assistance with financial issues and provision of medical supplies; case management; bereavement and grief support; and sibling support, including summer camp programs. Data was entered in a pass code protected access secured database, created and managed by the Evaluation staff person.

Statistical Packages for the Social Sciences (SPSS) and Excel software were used to perform descriptive analyses of results obtained from the HRQoL survey. Frequency and percentages of responses from the survey were calculated. Additional analyses were performed to assess the effects of length of Community PedsCare enrollment on HRQoL responses. A one-way analysis of variance (ANOVA) procedure was used to identify statistically significant differences in scores on the HRQoL for different lengths of Program enrollment. Regression analysis was performed to assess the relationship of HRQoL scores to period of enrollment.

### Hospital utilization and cost study

A secondary data analysis design was used to evaluate the impact of the Program on hospital utilization and costs. This outcome evaluation consists of two components: 1) a retrospective study of the utilization (length of stay) and costs (facility, laboratory, pharmacy, procedures, imaging, etc.) of services pre- and post-enrollment of children into the Program, and 2) comparison of hospital utilization and costs for Community PedsCare clients to utilization and costs prior to enrollment.

The retrospective study involved utilization and costs. Cost were categorized as follows: room and board, medical equipment and supplies, diagnostic costs, drug therapy, physical therapy, subspecialty institutional departments, pharmacy, dialysis, gastrointestinal services, and increment nursing. A purposeful sample of Community PedsCare clients who had documented hospital admissions during the 2 years prior to and the first two quarters after enrollment in the Program between the years 2002 to 2006 (*n* = 48) were included in the study. Because the client was referred based on referral criteria, the sample is more aligned with a purposive sample rather than a random sample of children.

Following Institutional Review Board (IRB) approvals, electronic data on patient conditions, types of care and costs were requested and obtained from Community Hospice of Northeast Florida Information Technology Department (Community PedsCare) and Baptist Health Information Services Department (Wolfson Children’s Hospital). The primary variables of concern included in the electronic data were: a) International Statistical Classification of Diseases (ICD 9 codes) of Community PedsCare clients, b) demographic data such as age, gender, etc., c) length of stay in the hospital, and d) hospital health care services and service related costs. Quarterly sums for hospital utilization and costs (facility, lab, pharmacy, procedures, imaging, etc.) were calculated per child for periods prior to and after enrollment into the Program.

The quarterly means for utilization and costs prior to enrollment in Community PedsCare were compared to the quarterly means for post enrollment periods. In order to ensure a comparable time frame for costs and utilization among clients who enrolled at various times, quarterly means of the variables of concern were only taken from Quarters 3 and 4 of 2005 and from Quarters 1 to 4 from 2006 before and after enrollment. The use of quarters provided large enough expenditures to be statistically reliable, and facilitate comparable time periods.

SPSS for Windows, release 15.0, Statistical Analysis System (SAS) Version 8.0, and Microsoft Office Excel 2003 software were utilized to test the primary hypotheses that Community PedsCare participation will be associated with decreases in utilization (length of stay in days) and hospital costs (facility and healthcare services charges). Datasets were linked and stripped of personal identifiers for confidentiality during analysis through SAS programming. Statistical significance was defined as differences at the .05 level, and for marginal statistically significant differences at the .20 level (important due to challenge of achieving statistical significance and avoiding Type II Error with very small sample). A paired *T*-test procedure was used to test for statistically significant differences in quarterly averaged hospital utilization and costs prior to and after enrollment into Community PedsCare among palliative care clients only.

## Results

### Health related quality of life

Fifty-three (*n* = 53) parents/caregivers participated in the HRQoL study. Participant demographics are presented in Table 1. Sixty-two percent (62 %) of participants in the HRQoL were White, 19 % were Black, and 79 % and 21 % of clients’ ages ranged from 0–12 years and 13–21 years respectively. The majority of clients (66 %) had been enrolled in the Program for more than 6 months.

**Table 1:** Demographics of Community PedsCare Pediatric Palliative Care Clients. Health Related Quality of Life Study, (2002–2007)

Client characteristics (*n* = 53)	Client frequency	Percent
Days in Pediatric Palliative Care:		
< 30 days	2	3.8 %
30 to 90 days	6	11.3 %
91 to 180 days	10	18.9 %
181 to 270 days	10	18.9 %
271 to 365 days	4	7.5 %
> 365 days	21	39.6 %
Gender:		
Female	29	54.7 %
Male	24	45.3 %
Race:		
Black	10	18.9 %
Hispanic	2	3.8 %
White	33	62.3 %
Native American	1	1.9 %
Unknown	7	13.2 %
Age Group:		
0–4 years	23	43.4 %
5–12 years	19	35.8 %
13–18 years	8	15.1 %
19–21 years	3	5.7 %
Family Caregiver Type of Pediatric Client:		
Father	3	5.7 %
Foster Parent	2	3.8 %
Grand Father	1	1.9 %
Grand Mother	2	3.8 %
Legal Guardian	2	3.8 %
Mother	42	79.2 %
Other	1	1.9 %

Data Source: Community Hospice of Northeast Florida Prepared by the Institute for Health, Policy and Evaluation Research

Overall, parents-caregivers tended to score high on HRQoL ordinal (1–5) and interval (0–30 days) scales for the HRQoL constructs/Items (i.e. decision-making, social support, interaction and communication, access to resources, child health). Table 2 displays results for all of the HRQoL constructs and related items. Participants reported excellent results on issues related to their capacity (self-efficacy) to care for their children, e.g., decision-making, meeting their child’s needs and managing their child’s health. For example, parents reported most or all the time:

**Table 2:** Health related quality of life survey results

Part I.						
HRQoL Constructs & Items	Responses by Range of Days					
General Emotional Health How many days during the past 30 days have you felt…	0 to 5 days	6 to 10 days	11 to 15 days	16 to 20 days	21 to 25 days	26 to 30 days
1.…stressed about your child’s health	18 (34 %)	11 (20.8 %)	5 (9.4 %)	2 (3.8 %)	0 (0 %)	17 (32.1 %)
2.…scared about your child’s health	37 (69.8 %)	6 (11.3 %)	1 (1.9 %)	0 (0 %)	0 (0 %)	9 (17 %)
3. …sad about your child’s health	25 (47.2 %)	8 (15.1 %)	3 (5.7 %)	2 (3.8 %)	0 (0 %)	15 (28.3 %)
4. …angry about your child’s health	45 (84.9 %)	3 (5.7 %)	2 (3.8 %)	0 (0 %)	0 (0 %)	3 (5.7 %)
5. …disappointed with my results when	46 (86.8 %)	3 (5.7 %)	2 (3.8 %)	0 (0 %)	0 (0 %)	2 (3.8 %)
Respite Care How many days in the last 30 days…						
6. …Was there someone to relieve you of your role of taking care of your child?	25 (47.2 %)	7 (13.2 %)	2 (3.8 %)	1 (1.9 %)	0 (0 %)	18 (34.0 %)
Activity Limitation How many days during the past 30 days …..						
7. …Were you not able to do your usual activities because of stress, depression, and other emotional problems	42 (79.2 %)	4 (7.5 %)	4 (7.5 %)	0 (0 %)	0 (0 %)	3 (5.7 %)
Part II.						
		Ordinal Scaled Responses				
HRQoL Constructs and Items		None of the time	A little of the time	Sometimes	Most of the time	All the time
Decision making		(1)	(2)	(3)	(4)	(5)
1. I am able to make good decisions concerning healthcare options for my child		0 (0 %)	0 (0 %)	1 (1.9 %)	13 (24.5 %)	39 (73.6 %)
2. I am able to find a way to make sure that my child has healthcare specific to their needs		0 (0 %)	0 (0 %)	5 (9.4 %)	13 (24.5 %)	35 (66.0 %)
3. I receive correct information about my child’s condition or illness		0 (0 %)	1 (1.9 %)	10 (18.9 %)	11 (20.8 %)	31 (58.5 %)
4. I feel confident in my decision to manage my child’s health		0 (0 %)	0 (0 %)	3 (5.7 %)	14 (26.4 %)	36 (67.9 %)
5. I am satisfied with decisions made for my child’s healthcare needs after a doctor’s visit		0 (0 %)	1 (1.9 %)	8 (15.1 %)	19 (35.8 %)	25 (47.2 %)
Social Support						
6. I have someone I can talk to about my fears concerning my child’s health		1 (1.9 %)	2 (3.8 %)	7 (13.2 %)	5 (9.4 %)	38 (71.7 %)
Interaction/Communication						
7. I can explain my child’s need to my primary healthcare provider		0 (0 %)	2 (3.8 %)	4 (7.5 %)	11 (20.8 %)	36 (67.9 %)
8. I can understand the needs of my child from my primary healthcare provider		0 (0 %)	0 (0 %)	4 (7.5 %)	16 (30.2 %)	33 (62.3 %)
9. I am able to ask questions I may have about my child’s healthcare		0 (0 %)	0 (0 %)	3 (5.7 %)	7 (13.2 %)	43 (81.1 %)
10. My child has someone they can express themselves to when they are sad, angry, afraid, etc…		9 (17.0 %)	0 (0 %)	3 (5.7 %)	4 (7.5 %)	37 (69.8 %)
Access to Resources						
I am able to obtain or have assistance in obtaining the following:						
11. Medicine		1 (1.9 %)	0 (0 %)	2 (3.8 %)	6 (11.3 %)	44 (83.0 %)
12. Medical equipment		0 (0 %)	0 (0 %)	6 (11.3 %)	13 (24.5 %)	34 (64.2 %)
13. Housing and Utilities		4 (7.5 %)	0 (0 %)	3 (5.7 %)	4 (7.5 %)	42 (79.2 %)
Child Health						
14. I am able to understand the needs of my child		0 (0 %)	0 (0 %)	3 (5.7 %)	16 (30.2 %)	34 (64.2 %)
15. My child understands their condition		29 (54.7 %)	2 (3.8 %)	7 (13.2 %)	3 (5.7 %)	12 (22.6 %)
16. My child spends quality time with family and friends		1 (1.9 %)	0 (0 %)	4 (7.5 %)	5 (9.4 %)	43 (81.1 %)
17. My child is treated with dignity while receiving healthcare services		0 (0 %)	0 (0 %)	1 (1.9 %)	4 (7.5 %)	48 (90.6 %)

- ▪ The ability to make good decisions concerning health care options for their child (94 %), and confidence in their ability to manage their child’s health (94 %).
- ▪ Being able to ask questions to their health care providers about their child’s healthcare needs (94 %), and understanding their responses (93 %).
- ▪ Being able to obtain medicine required for their child’s health needs (94 %).
- ▪ Understanding the needs of their child (94 %), providing their child quality time with family and friends (91 %), and receiving ethical healthcare (98 %).

Parents reported that within the last 30 days, perceptions of: a) impaired emotional health averaged 7 days, b) activity limitations due to impaired emotional health averaged 8 days, and c) relief from care giving averaged 13 days.

Parental perceptions related to externally controlled issues, in particular physician services and health system functions were also rated highly, but lower than the internal locus of control items. For instance, a substantial number of parents reported that only sometimes, a little of the time or none of the time, they:

- ▪ Were able to find a way to make sure that their child had health care specific to their needs (9.4 %); received correct information about their child’s condition or illness (21 %); and were satisfied with decisions made by their child’s doctor (17 %).
- ▪ Have someone to talk to about fears concerning their child (19 %).
- ▪ Can explain their child’s needs to their primary healthcare provider (11 %).
- ▪ Are able to obtain or have assistance in obtaining medical equipment (11 %) and housing and utilities (13 %).

With respect to children themselves, parents reported some, a little or none of the time that their children: a) had someone with whom they could express themselves when they are sad, angry, afraid, etc. (23 %), and b) understand their condition (72 %). This could be attributed to the proportion of children less than 5 years of age (43.4 %) and children with developmental and cognitive impairments.

**Fig. 2:**
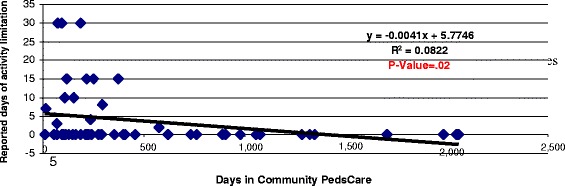
Relationship of PedsCare period of enrollment to activity limitation due to adverse emotional health

**Fig. 3:**
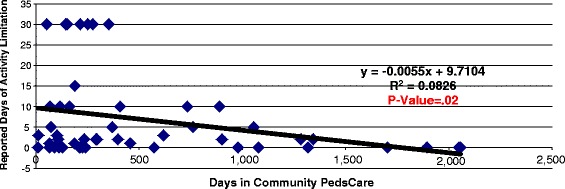
Relationship of PedsCare period of enrollment to days of feeling scared about child’s health

Additional analysis using Analysis of Variance (ANOVA) was conducted to assess the relationship of length of enrollment in PedsCare to the HRQoL responses. The analysis identified significant (*p* ≤ .05) differences in reported days of impaired emotional health due to fear (*p* = .01) and differences in reported days of activity limitation due to emotional problems (*p* = .01) associated with the length of enrollment. An additional regression analysis identified statistically significant linear relationship for the length of enrollment to HRQoL scores related to reduced fear (*p* = .02) and reduced activity limitations (*p* = .02) with the regression charts showing responses for days of limitation in a month (30 day period) for children enrolled over a continuum or periods from zero to almost 6 years of the program (approximately a 1500 day maximum enrollment period). [See Figs. 2 and 3].

### Utilization and cost

**Fig. 4:**
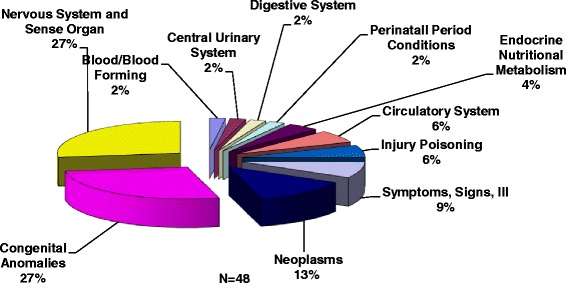
Diagnosis Group of Parent/Guardian’s child enrolled in pediatric palliative care while utilizing hospital services (2000–2006)

Children enrolled in Community PedsCare through the years 2000 and 2006 were eligible for inclusion in the utilization and cost study. The illnesses and conditions of children enrolled in the Program during these years are presented in Fig. 4. The total cost of the 1440 regional hospital admissions (2000–2006) of children (enrolled and not enrolled in the Community PedsCare program) with the diagnoses of children in the Community PedsCare program was $56,626,703.

Table 3 presents the demographics of Community PedsCare clients (*n* = 48) included in the hospital utilization and cost studies. From 2000 to 2006, 58 % of Community PedsCare palliative care clients utilized hospital services before and after enrollment in the Program, and 42 % were not hospitalized while in the Program. The majority of clients utilizing inpatient hospital services was White (67 %) and had the following conditions: Congenital Anomalies (28 %), Nervous Organs/Sense Organs (27 %) and Neoplasms (13 %). See Table 3.

**Table 3:** Demographics of Community PedsCare clients* (2000–2006). Cost and Utilization Study

Client characteristics (*n* = 48)	Client frequency	Percent
Client palliative care enrollment status:		
Number of clients enrolled in Community PedsCare who utilized the hospital post enrollment	28	58 %
Number of clients enrolled in Community PedsCare who were not hospitalized post enrollment	20	42 %
Days in the program as of 7/1/07:		
180-365	18	38 %
>365	30	62 %
Gender:		
Female	32	67 %
Male	16	33 %
Race:		
Black	9	19 %
White	32	67 %
Hispanic	6	13 %
Other	1	2 %

Data Source: Community Hospice of Northeast Florida Prepared by the Institute for Health, Policy and Evaluation Research

Results from the utilization analysis show statistically significant differences for Community PedsCare patients who utilized hospital services during the pre-enrollment quarters compared to the quarters following their enrollment. In order to ensure a comparable time frame for costs and utilization, quarterly means of the variables of concern were only taken from Quarters 3 and 4 of 2005 and from Quarters 1 to 4 from 2006 before and after enrollment. Table 4 reveals that prior to enrollment, Community PedsCare clients’ length of stay in the hospital averaged 2.92 days per quarter. After enrollment in the Program, client length of stay in the hospital significantly decreased to an average of 1.22 days per quarter (*p* value < .05).

**Table 4:** Comparison of hospital utilization and costs: before and after pediatric palliative care, PedsCare clients only

Community PedsCare pediatric client	Before pediatric palliative care		After pediatric palliative care		*P*-value*
	*N* = 40		*N* = 40		
Cost and utilization findings	Mean	Standard error	Mean	Standard error	
Length of Stay (Days/Quarter)	2.92	.94	1.22	.39	0.03
Total Diagnostic Charges/Quarter	$2,125.30	918.44	$1,078.28	430.11	0.13
Total Charges/Quarter	$7,866.59	2,347.31	$6,663.52	2,785.22	0.34

*Paired One tailed *T*-test -Before and After Community PedsCare

Data Source: Baptist Health System: Baptist Medical Center Downtown

Prepared by Duval County Health Department, Institute for Health, Policy, and Evaluation Research

Following enrollment in the Program, hospital charges declined by $1203 for total charges per quarter for hospital services and $1047 for diagnostic charges per quarter. The decrease in diagnostic charges was marginally significant (*p* = 0.13). Although the total charges decreased, the decline did not reach statistical significance (*p* = 0.34).

## Discussion

The Medical Home model was pioneered five decades ago by the American Academy of Pediatrics to improve the care of children with special health care needs [36, 37]. Subsequent work by Cal Sia, M.D. and others established the principles of practice and policies that have advanced the model to become the Patient-Centered Medical Home embraced by all primary care specialties [36, 38]. Concurrent development of the practice of Community Pediatrics by Robert Haggerty, M.D. established the importance of engaging community resources to expand the services available to primary care providers required by their patients—in particular children with special health care needs [39]. Despite the decades-old development of these evidence-based practices, the practice of pediatric hospice and palliative medicine and community-based pediatric palliative care are less than a decade old and relatively few communities have access to these services. In addition, palliative care services remains unreimbursed severely limiting the expansion of these services [40]. Thus, it is imperative that an evidence-base be generated to validate the effectiveness of community-based palliative care to improve the health and well-being of children with chronic complex medical conditions and decrease the cost of care as a complement to the patient-centered medical home.

Toward this end, multiple regional and national efforts are unfolding to improve quality of care of children with chronic complex-medical conditions, driven primarily by efforts to decrease the cost of their care [41]. Although many of these endeavors focus on developing enhanced patient-centered medical homes and more effective hospital-based care for children with complex-medical conditions, few if any of these initiatives include community-based palliative care as a core element of their medical home strategies. This is in part due to the failure of private sector insurance to cover community-based palliative care services. Several states have Medicaid waivers that cover some of these in-home services, but reimbursements are meager and services limited—and there have been few examples of such programs in the private sector. The Affordable Care Act provides an opportunity through its *Concurrent Care* program to provide concurrent curative and community-based hospice care to children, but children must be eligible for hospice in order to participate [42].

The decrease in hospital utilization and costs for children post enrollment in the Community PedsCare program, the positive perceptions of health related quality of life related to enrollment in Community PedsCare, and the relationship between length of enrollment in Community PedsCare and quality of life for several HRQoL domains indicate that community-based pediatric palliative care could play a defining role in expanding the structure of patient-centered medical homes, the holistic care of children with complex medical conditions, and the function of pediatric health care systems in response to the increasing number of children with chronic complex medical conditions [25].

Given that it is reasonable to expect that the health status of children in the Community PedsCare Program declined over time, the Program’s potential impact on decreasing hospital utilization and cost post-enrollment in the Program may be even more significant. These findings, and the tendency for caregivers to report high HRQoL scores, could inform insurance companies and other payers of the potential benefits of expanding coverage to include community-based pediatric palliative care services. Moreover, these findings indicate the potential value of empowerment and the quality community-based support in care planning process [43]. Demonstrating potential for improved quality of life and decreased costs of care, as reported in this study, will be necessary to legitimize and catalyze comprehensive public and private sector third-party reimbursement for community-based palliative care.

### Strengths, limitations, and lessons learned

Important lessons were learned about the feasibility and limitations of several approaches to the research methods used in this initial study, which can inform development of future research.

- ▪ The small sample size was a major factor in limiting conclusions. Future impact assessments of pediatric palliative care will be substantially enhanced through longitudinal studies at multiple sites that could include larger samples and samples of children with very similar conditions.
- ▪ Comparison of hospital utilization and cost pre- and post-enrollment in a pediatric palliative care program is a viable approach to determining impact, though the disease course will negatively impact cost savings, as presumably the child’s clinical status will worsen over time. Any positive impact on utilization and cost, and other illness and condition-related system of care variables, will be somewhat moderated by the condition’s course. Even a modestly positive impact should therefore be interpreted as a significant gain.

ICD 9 codes and other disease classifications, e.g. Clinical Risk Groups (CRGs) alone are not adequate for identifying appropriate comparison groups for children enrolled in pediatric palliative care programs, as these codes do not adequately address severity of illness, a major factor in service utilization and cost. Presumably the most ill children within a coding group will more likely be enrolled in a palliative care program and/or receiving palliative care services. Future studies may need to consider implementation of additional resources that identify the most common, specific diagnoses found in pediatric palliative care [44].

- ▪ Comparing children receiving palliative care services with those who are not, and/or comparing those enrolled and not enrolled in palliative care programs may not result in the comparison of comparable groups. An additional approach to clarifying severity of illness is necessary to identify comparison groups to assess the impact of palliative care services and programs.
- ▪ Dose effect (the amount of time after enrollment required to produce an effect) may need to be determined before palliative care service and program impact can be fully assessed. Future impact assessments, in particular those related to HRQoL domains, should involve longitudinal data collection beginning at the time of enrollment and at standardized periods thereafter to assess the impact of palliative care services and programs.
- ▪ This evaluation focused only on hospital utilization and costs. The impact of pediatric palliative care services and programs on other system-related variables, e.g. the number of hospitalizations over time; and emergency department, outpatient, subspecialty, etc. utilization and costs represent important areas for future research.
- ▪ This study did not provide a cost-benefit analysis. The potential benefits of the program that were demonstrated by this study were not analyzed related to the costs of providing the services.
- ▪ The HRQoL assessment tool was an initial effort to quantify relevant health related quality of life factors. It primarily reflected HRQoL benefits from the perspectives of program professionals. The instrument requires more extensive validation and ongoing refinement to increase discrimination power and address the perspectives of family members.
- ▪ HRQoL questions focused on children must be analyzed in the context of the child’s chronological age and developmental and cognitive capacities.
- ▪ Pre and post enrollment assessment of HRQoL will also need to be conducted to more accurately assess program impact over time.

Though the sample size was small, the results of this study show promising results. Future studies will require larger sample sizes, a longitudinal approach, consideration of the effects of length of enrollment on outcomes and other methodological refinements.

Pediatric palliative care by its nature is responsive to the perceived needs and concerns of patients and families. As such, it can inform ACA catalyzed health service research, system reforms and policy development to advance the relevance of the Patient Centered Medical Home to the care of children with chronic complex medical conditions [45]. Despite the global development of pediatric palliative care programs, only a minority of children in need receive this type of care [9]. In order to expand the practice of pediatric palliative care, it is imperative that future research generates an evidence base that informs the: a) care and provision of services for children with chronic complex medical conditions, b) structure, function and finance of pediatric health care systems that advance the *pediatric palliative care continuum*, including its integration into the patient-centered medical home model, and c) curricula and pedagogy for the interdisciplinary training of child health professionals in palliative care.

## Conclusion

This pilot study demonstrates the potential impact of CBPPC on improved HRQoL and decreased cost of care. The HRQoL results showed parents-caregivers’ reported overall positive perceptions with impaired emotional health, decision-making, social support, interaction and communication, child health and self-efficacy in caring for their children, with higher HRQoL scores associated with longer periods of enrollment. The utilization analysis showed reductions in utilization of hospital services for Community PedsCare patients during the pre-enrollment quarters compared to the quarters following their enrollment. This evaluation was among the first and remains among the few in the US to assess the impact of community-based pediatric palliative care on health related quality of life and hospital utilization and costs [7]. This pilot study yielded promising results and suggests the need for further investigation.

## Abbreviations

ACGME, Accreditation Council for Graduate Medical Education; ANOVA, Analysis of Variance; CBPPC, Community Based Pediatric Palliative Care; CRG, Clinical Risk Group; HRQoL, Health Related Quality of Life; ICD, International Classification of Disease; IRB, Institutional Review Board, SAS, Statistical Analysis System; SPSS, Statistical Packages for the Social Sciences

## Additional file

Additional file 1: Community PedsCare A Summative Evaluation of a Pediatric Palliative Care Program. Final Report. (PDF 16085 kb)
