# Erratum to: Community-based pediatric palliative care for health related quality of life, hospital utilization and costs lessons learned from a pilot study

**DOI:** 10.1186/s12904-016-0154-z

**Published:** 2016-09-15

**Authors:** Jeffrey Goldhagen, Mark Fafard, Kelly Komatz, Terry Eason, William C. Livingood

**Affiliations:** 1 Division of Community and Societal Pediatrics, Department of Pediatrics, UF College of Medicine – Jacksonville, 841 Prudential Drive, Suite 1330 m, Jacksonville, FL 32207 USA; 2 Baptist Health Research Institute, Baptist Health System, 836 Prudential Drive, Pavilion 6th Floor, Jacksonville, FL 32207 USA; 3 Community PedsCare, Community Hospice of Northeast Florida, 4266 Sunbeam Rd., Jacksonville, FL 32257 USA; 4 Center for Health Equity and Quality Research, UF College of Medicine-Jacksonville, 580 W. 8th St., Tower II, Room 6015, Jacksonville, FL 32209 USA

## Erratum

Upon publication of the original article [1], minor errors were noticed in the Article Title. This has now been corrected in this erratum. Instead of:

“Community-based pediatric palliative care for health related quality of life, hospital utilization and costs lessons learned from a pilot study”

The Title should read:

“The impact of community-based palliative care on health related quality of care, hospital utilization and costs: Lessons learned from a pilot study”
