# Supplementary material for: Community-based pediatric palliative care for health related quality of life, hospital utilization and costs lessons learned from a pilot study
Source: BMC Palliat Care. 2016 Aug 3;15:73. doi: 10.1186/s12904-016-0138-z (PMC4971636; doi:10.1186/s12904-016-0138-z)
Supplement: Additional file 1: — Community PedsCare A Summative Evaluation of a Pediatric Palliative Care Program. Final Report. (PDF 16085 kb) [file 12904_2016_138_MOESM1_ESM.pdf]

Community PedsCare

A Summative Evaluation of a Pediatric Palliative Care Program

Final Report

Submitted by:

The Institute for Health, Policy and Evaluation Research
Jacksonville, Florida
March 2008

Authors

Jeffrey Goldhagen, M.D., MPH
William Livingood, Ph.D.
Kimberly Pierce, MPH
Thomas Bryant III, MSW

Acknowledgements: Eulisa Morgan-Murphy, BS, Anita Davis, BA, and the Community PedsCare Team

Table of Contents

I. **Executive Summary**.. **3**
II. **Introduction**.. **7**
A. Background.. 8
III. **Evaluation Methods**.. **10**
A. Hypothesis.. 10
B. Study Design.. 10
IV. **Cost and Utilization Study**.. **11**
A. Cost and Utilization Design.. 11
1. Cost and utilization measures 11
2. Comparison of hospital utilization and costs before and after program enrollment, Community PedsCare clients only.. 11
3. Comparison of Pediatric Palliative Care Clients vs. Control Group........ 12
4. Analysis 13
B. Results 13
1. Demographic Profile of Pediatric Clients for Cost and Utilization Study.. 14
2. Cost and Utilization outcome findings 15
V. **Health Related Quality of Life Study**.. **17**
A. Health Related Quality of Life Design.. 17
1. Purpose and Instrument Development.. 18
2. Data collection.. 18
3. Analysis.. 18
B. Results 18
1. Demographic Profile of Pediatric Clients for Health Related Quality of Life Study.. 19
2. Health Related Quality of Life outcome findings 26
VI. **References**.. **31**

Appendix A- Evaluation Logic Model

Appendix B. Health Related Quality of Life Survey

Appendix C. Data Dictionary

Appendix D. Community PedsCare Represented Illnesses

Appendix E. Quality Improvement: Process Measures

Community PedsCare

A Summative Evaluation of a Pediatric Palliative Care Program

EXECUTIVE SUMMARY

PURPOSE: The purpose of this project was to evaluate the Community PedsCare pediatric palliative and hospice care program. This evaluation was intended to provide an assessment of the impact of PedsCare palliative care services on hospital utilization and costs, and parental perceptions of health related quality of life (HRQOL). In addition, as few previous studies have been conducted and reported, new evaluation methods and tools were to be pilot tested and evaluated.

CONTEXT: Children with chronic illnesses that are life threatening or limiting present major challenges for medical professionals who are faced with balancing aggressive medical treatments with quality of life issues for both children and their families. Increasing numbers of children are living with these illnesses as a result of rapidly expanding medical expertise and technology. In response to these challenges, Community PedsCare was established a decade ago to coordinate community-based services for children with life threatening and limiting conditions and their families. The program was established as a collaboration between Wolfson Children's Hospital, Community Hospice of Northeast Florida, Nemours Children's Clinic, the University of Florida College of Medicine-Jacksonville and the community. In January 2007, Community Hospice and Baptist Health Foundation partnered with the Duval County Health Department's Institute for Health, Policy and Evaluation Research and University of Florida to conduct the first summative evaluation of the PedsCare program, with funding from the Jessie Ball duPont Fund.

METHODS: This outcome evaluation consists of three components: 1) a retrospective study of the cost and utilization of services pre- and post enrollment of children into the program; 2) comparison of the utilization and cost for PedsCare clients with other children with similar ICD 9 coded conditions; and 3) a retrospective study to assess the perceived health related quality of

life (HRQOL) of family caregivers of PedsCare clients. The utilization and cost studies relied on hospital records of cost and utilization for PedsCare clients and other children who were hospitalized with similar conditions as reflected in comparable ICD 9 codes. The HRQOL study involved the development and use of a HRQOL instrument, and interviews with PedsCare family members and care takers.

RESULTS: There were 1,440 hospital admissions to Wolfson Children's Hospital (2000 to 2006) for children with illnesses cared for by Community PedsCare (Appendix D for represented illnesses). Among the 1,440 admissions, congenital anomalies (257.6 per 1000 pediatric hospital admissions), injuries (195.8 per 1000 pediatric hospital admissions), and malignant neoplasms (81.9 per 1000 pediatric hospital admissions) represented the three most commonly diagnosed illnesses. The total cost for the 1,440 hospital admissions from 2000 to 2006 was $56,626,703. There was a significant decrease ($p = .03$) in hospital utilization (length of stay) for clients after enrollment into Community PedsCare, in comparison to their pre-enrollment utilization. There was a promising decline in service charges post-enrollment, but the decline was not statistically significant ($p = .34$). No significant differences were identified in service utilization and costs between PedsCare clients and non-PedsCare children with similar ICD 9 codes. This may have resulted from the inadequacy of ICD 9 codes to reflect the severity of illnesses. This would negatively impact the validity of this methodological approach. The HRQOL study revealed that family caregivers tended to score high on the HRQOL scales, but nevertheless did report a number of days of impaired functioning in the last 30 days. HRQOL scores for issues related to the self-efficacy of family/caregivers to care for their children tended to be higher than those that reflected the function of the health care system. Analysis of parental responses to the HRQOL survey revealed significant ($p \leq .05$) and marginally significant ($p \leq .20$) relationships to clients' length of enrollment in the Community PedsCare for the following: 1) reported days of impaired emotional health due to fear ($p = .01$); 2) reported days of impaired emotional health due to sadness ($p = .16$); and 3) reported days of activity limitation due to emotional problems ($p = .01$).

CONCLUSIONS: This is among the few, and may be the first utilization and cost and HRQOL evaluation of pediatric palliative and hospice care in the U.S. The evaluation shows promising results related to reduced hospital utilization and costs, as well as improved quality of life for

children and families enrolled in the PedsCare program. The comparison study did not reveal a statistically significant difference between PedsCare clients and a control group. The results are preliminary due to: 1) the small overall sample size; and 2) methodological challenges encountered in using ICD 9 codes to identify comparable PedsCare and non-PedsCare groups (types and severity of conditions) of children for the impact study. Caution should be exercised in interpreting the results, but further study is clearly warranted. Longitudinal and multi-site investigations using the tools and approaches piloted in this study should be implemented.

RECOMMENDATIONS: Based on these results, the following recommendations are provided for future studies:

- The small sample size was a major factor in limiting conclusions. The impact assessment will be substantially enhanced if the evaluation is expanded to include all children enrolled in the PedsCare program in the future, and if it is extended to include multiple sites.
- Comparison of hospital utilization and cost pre- and post-enrollment into the Program is a viable approach to determining impact, though the disease course will negatively impact cost savings, as presumably over time the child's clinical status will worsen. Any positive impact on these types of variables will be somewhat moderated by the disease course. Even a modestly positive impact should therefore be interpreted as a significant gain.
- ICD 9 codes alone may not be adequate for identifying an appropriate comparison group for children enrolled in the PedsCare program, as the ICD 9 codes may not adequately address severity of illness, a major factor in service utilization. Presumably the most ill children within an ICD 9 coding group will be in palliative care, so comparing children in and not-enrolled in a palliative care program may not result in the comparison of comparable groups. An additional approach to clarifying severity of illness is necessary to identify a comparison group to assess impact.
- Dose effect (the amount of time after enrollment required to produce an effect) may need to be determined before impact can be fully assessed. Future impact assessments could involve data collection with the HRQOL prior to client enrollment and at a standardized time after enrollment to assess the impact of the program.

- The HRQOL survey was developed using multiple "experts" and families. In general parents/caregivers scored high on questions. Revisions will need to be made over time with feedback from families and professionals to improve its sensitivity and validate the instrument.

Quality Improvement processes and measures that facilitate identifying the relationship between enrollment in PedsCare and outcomes (e.g., cost, utilization, quality of life, etc.) should be continued to enhance both the impact and quality of the program.

INTRODUCTION

Children with life threatening and limiting chronic illnesses present major challenges for medical professionals faced with balancing aggressive medical treatments with quality of life for both children and families. In response to this challenge, pediatric palliative care is a newly emerging specialty which has evolved from a focus on end-of-life care to care emphasizing relief of suffering and improvement of quality of life (Himelstein, 2006; Weidner, 2006; Gunten, 2005). Few studies have reported the effectiveness of this care and its impact on major outcomes, such as hospital utilization and cost.

Palliative care principles go beyond biomedical science to include communication techniques of physicians and healthcare providers that impact health related quality of life outcomes (Tulsky, 2005, Contro et al, 2002). Past studies have shown that physicians and other health care providers do not sufficiently discuss treatment options or respond to emotional issues of concern to patients and families (Tulsky 2005, Contro et al 2002; Sourkes, 2005). Other issues of concern for families and patients with healthcare providers include decisions made for the child's healthcare, inadequate support, and unnecessary suffering (pain and symptom management) due to curative therapy (Contro et al. 2004). Evidence shows that pediatric palliative services that focus on appropriate communication, decision support, and case management in a home environment have the potential to improve quality of life of the family and pediatric client (Hayes 2006; Hynson and Sawyer, 2001; Meier, 2007). While principles are emerging, well defined palliative care models with evidence of effectiveness have not been reported. Another major issue of concern, cost effectiveness of pediatric palliative services, has not been proven due to lack of data (Davis 2006, Himelstein 2006 and Morrison 2005).

This report provides findings of an evaluation study designed to develop and pilot data collection and analysis methods to assess the impact of a palliative care program designed to improve the quality of life and well-being of chronically ill children and their family members, and decrease hospital cost and utilization. The dearth of documented evidence in the literature of the impact of pediatric palliative care on outcomes enhances the importance of this local evaluation.

Background

Before an assessment of the Community PedsCare program was conducted, an analysis was performed to document the number of children admitted into Wolfson Children's Hospital with the illnesses represented in Community PedsCare (Appendix D). Among the 1,440 admissions, congenital anomalies (257.6 per 1000 pediatric hospital admissions), injuries (195.8 per 1000 pediatric hospital admissions), and malignant neoplasms (81.9 per 1000 pediatric hospital admissions) represented the three most commonly diagnosed illnesses (Figure 1). The total costs for the 1,440 hospital admissions from 2000 to 2006 was $56,626,703. The length of stay varied extensively among these conditions (Figure 1).

Figure 1

Comparison of Length of Stay and Rate of PedsCare Represented Diagnoses for Pediatric Inpatient Hospital Admissions(n=1,440), Northeast Florida, (2000-2006)

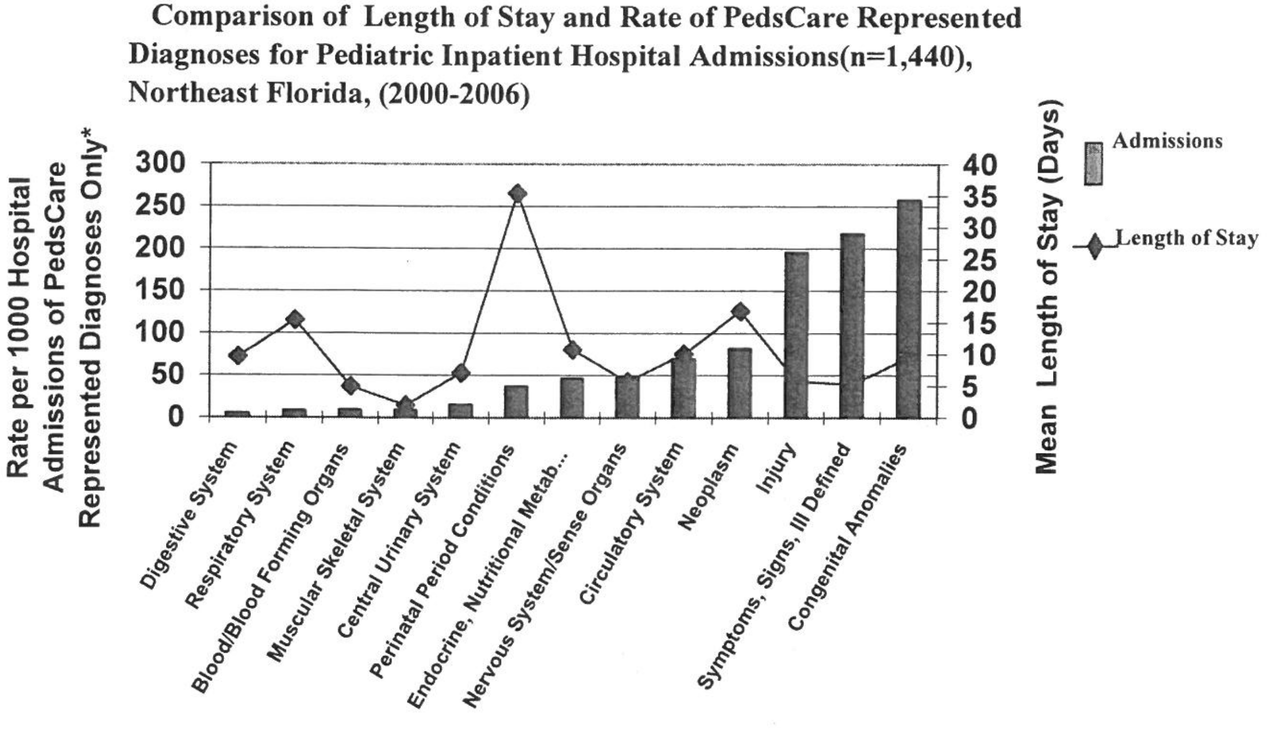

*Total Hospital Admissions of PedsCare Represented Illnesses Only
Source: Florida Department of Health, Agency for Health Care Administration, 2000-2006
Prepared by: DCHD, Institute for Health, Policy and Research Division

Figure 1 illustrates the disproportionate demand on hospital utilization (length of stay) for some conditions represented in PedsCare in Northeast Florida. Undoubtedly, some conditions require much more extended duration of treatment. Because medical care is increasingly extending the lifespan of children without curing their diseases, these children are likely to experience a lifetime of extensive medical care The high probability of a lifetime of debilitating disease without cure has created a growing consensus that quality of life for these children and their families should be a priority. Palliative care has emerged as the approach to providing care in a manner that optimizes quality of life for children with chronic illnesses and their families.

In an effort to address the need for care coordinated services for children with chronic illnesses, Community Hospice of Northeast Florida collaborated with Wolfson Children's Hospital, Nemours Children's Clinic, University of Florida College of Medicine-Jacksonville and the community to establish Community PedsCare in September 2001. The program cares for children under the age of 21, regardless of their financial status or insurance coverage, to improve the quality of life for children with debilitating chronic illness and their families. In January 2007, PedsCare established a close collaboration with Children's Medical Services (CMS) to become their provider of palliative care services for children enrolled in their Partners in Care (PIC) program. Partners In Care is a Medicaid waiver program that provides reimbursed multi-disciplinary palliative care services for children, siblings and families.

The core palliative care services offered by Community PedsCare include:

- Palliative care home visits by physicians, nurses, social workers, a child life therapist, a chaplain and volunteers.
- Palliative care consults in homes and hospitals by a physician.
- Medical supplies for palliative care patients who have no other provision or payor source.
- Bereavement support and counseling to the sick child and their family members.
- Psychosocial support to the sick child's teachers and classmates.

EVALUATION METHODS

In January 2007, Community Hospice and Baptist Health Foundation partnered with the Duval County Health Department's Institute for Health, Policy and Evaluation Research and the University of Florida College of Medicine-Jacksonville, with support from the Jesse Ball duPont Fund, to conduct the first summative evaluation of the Community PedsCare program. This evaluation was intended to provide an assessment of the impact of palliative care services on hospital utilization and costs, and parental perceptions of health related quality of life (HRQOL). In addition, as few previous summative studies of pediatric palliative care have been conducted and reported in the U.S., new evaluation methods and tools were to be pilot tested and evaluated.

Hypotheses

The primary hypotheses are:

1. Community PedsCare clients will have significantly lower hospital utilization and costs following enrollment into the palliative care program.
2. Community PedsCare clients will have significantly lower hospital utilization and costs than pediatric patients in a comparison group.
3. Community PedsCare family/caregivers achieve higher health related quality of life following enrollment into the palliative care program.

Study Design

This evaluation is intended to develop a study design that will provide a clear evidence-based approach that can be adaptive and sensitive to the evolving nature of pediatric palliative care programs in general, and the Community PedsCare program in-particular. This approach includes:

1. The use of a logic model to identify potential outcomes (intermediate and distal) experienced by the pediatric client and the family/caregiver in the healthcare system (Appendix A).
2. The use of hospital data and a control group to assess program impact on hospital utilization and costs.

3. The development of a HRQOL assessment tool to measure the impact of pediatric palliative care on health related quality of life (Appendix B).

COST AND UTILIZATION STUDY

A retrospective design was used to document the Community PedsCare impact on hospital utilization and costs of pediatric palliative care clients. Two comparisons were conducted to assess program impact on utilization and costs: 1) utilization and costs were compared before and after client enrollment into the PedsCare program, and 2) utilization and costs for the PedsCare clients after enrollment were compared to similar measures for a control group.

Cost and Utilization Measures

Electronic data were obtained from Community Hospice of Northeast Florida Information Technology Department (PedsCare) and Baptist Downtown Medical Center Information Services Department (Wolfson Children's Hospital). The primary variables of concern that were included in the electronic data were:

1. International Statistical Classification of Diseases (ICD 9 codes) of PedsCare clients,
2. Demographic data such as age, gender, etc.,
3. Length of stay in the hospital, and
4. Hospital healthcare service charges.

Comparison of Pediatric Palliative Care Before and After Program Enrollment, Community PedsCare Client Only

The intervention group, identified through purposeful sampling, consisted of Community PedsCare clients who were enrolled in the palliative care program from 2002 to 2006 (n=48). In order to measure the impact of hospital utilization and costs before and after enrollment in PedsCare, PedsCare clients' hospital utilization and cost data were obtained from Baptist hospital's Information Services. Quarterly sums for hospital utilization (length of stay) and costs were calculated for periods prior to enrollment into the program and periods after

enrollment in the program for Community PedsCare clients. The quarterly means for costs and utilization prior to enrollment were compared to the quarterly means post enrollment periods.

Sampling: Community PedsCare clients who utilized the hospital prior to and after enrollment in the program were purposefully selected based on the following selection criteria:

1. Hospital utilization during the previous two years prior to enrollment in the Community PedsCare palliative care program.
2. Hospital utilization during the first two quarters after enrollment in the Community PedsCare palliative care program.

Since Baptist hospital data were the only data available to determine pre-enrollment costs and utilization, data for only 43 of the 48 PedsCare clients were available for both pre- and post-periods. Five of the clients had not received care at Baptist prior to PedsCare enrollment. Three others were excluded because of exceptionally high use (outliers), reducing the number of clients to 40 for the before and after analysis.

Comparison of Pediatric Palliative Care Clients vs. Control Group

Sampling: A criterion of hospital utilization during the previous 6 months was used for selection of the PedsCare sample to ensure a minimum exposure effect necessary to assess program impact. ICD 9 codes were identified from Community PedsCare Clients and used to identify eligible clients for the control groups. Utilization and cost variables were identified and obtained for both PedsCare palliative care clients and the control group. The control group was purposefully selected based on the following criteria:

1. Less than 21 years of age.
2. A complex chronic condition (ICD 9 code) identical to PedsCare pediatric client.

A 1:2 matching procedure was performed for selection of the control group for comparison to PedsCare clients, using a SAS Macro language and confirmed through a cross tabs procedure performed in SPSS to identify non PedsCare clients with similar ICD 9 codes and admission dates comparable to PedsCare clients. In order to ensure a comparable time frame for costs and

pediatric palliative care. The logic model presented in Appendix A provides a framework to support and ensure consistency of future studies.

Community PedsCare was established by Community Hospice of Northeast Florida in 2000 to meet the needs of children and families in Northeast Florida facing life threatening and limiting illness. The program is a collaboration with Wolfson Children's Hospital, Nemours Children's Clinic, the University of Florida College of Medicine–Jacksonville, Children's Medical Services, the Jesse Ball duPont Fund and the community. The program works in collaboration with physicians and other professionals and organizations to ensure children and families receive care designed to relieve suffering, provide comfort and improve the quality of all aspects of a child's life and that of their family's. Program professionals include medical, nursing, mental health, social service, child life and clergy. Current services include pain and symptom management; medical consultation; mental health, psychosocial and spiritual support and counseling; family respite; assistance with financial issues; case management; and bereavement and grief support. Special attention is paid to the needs of siblings.

The statistically significant decrease in hospital utilization and the trend in decreased costs for children pre- and post enrollment in the Program indicate that pediatric palliative care could play a defining role in restructuring a pediatric health care system—a system that will need to respond to the increasing number of children with special health care needs. Given that it is reasonable to expect that the health status of children in the PedsCare program declined over time, the Program's impact on decreasing utilization and cost post-enrollment in the Program is even more significant. These findings could inform insurance companies and other payors of the potential benefits of expanding coverage to include pediatric palliative care services. Currently few such benefits exist, which has severely limited the growth and sophistication of community-based pediatric palliative care. Programs that do exist have been limited to a paradigm that has tended to mimic the structure and function of adult hospice care and benefits, unless philanthropic sources of support can be identified to sustain community-based services and care.

The PedsCare program studied in this evaluation is primarily a community-based palliative care service. Nationally, most pediatric palliative care programs have developed as hospital-

based programs or as separate community-based services. There are few comprehensive programs that integrate community and hospital-based services as a continuum of care. Though the Jacksonville community is currently in the process of developing a fully integrated program, these evaluation results do not reflect the status of this emerging program. It is reasonable to project that programs that integrate hospital and community-based services will have an even greater impact on hospital utilization and costs, and health related quality of life. This is important, as the future architecture of pediatric palliative care is currently being established primarily as hospital-based services. The capacity of a community-based service in and of itself to positively impact hospital utilization and cost and quality of life, as demonstrated in this study, should establish the integration of community and hospital-based services as the benchmark for future programs.

The promising results of this study reflect the changing epidemiology of children being served by pediatric palliative care services in Jacksonville and across the country. The causes of childhood illness and death have changed dramatically over the past several decades. Advances in immunizations, antibiotics, genomics, new drugs and medical technology have changed the landscape of children's health. We are now faced with a new set of challenges—how to respond to the increasing burden of chronic illness in children, its impact on families and communities, and the delayed trajectory of dying that is prolonged by advances in medicine. Pediatric palliative care is essentially a chronic disease model of care for children along the continuum of chronic illness. It makes sense that a model of care for children with chronic illnesses in which the locus of service is in the home and community, as opposed to the hospital, will have positive impacts as demonstrated in this study.

To be optimally effective from a cost and quality perspective, emerging models for pediatric palliative care must provide a continuum of community-based medical, psychosocial and spiritual support services to children and families facing life-limiting and life-threatening illnesses, linked to hospital-based care. These community-based services must: a) manage symptoms and relieve physical and emotional distress produced by medical conditions, b) help children, siblings and extended families live as normally as possible and improve their quality of life, and c) provide timely and accurate information to support children, families and caregivers

in decision making. When linked to hospital-based palliative services, the pediatric palliative care continuum establishes a comprehensive approach to the care of children with chronic illnesses and their families.

The results of the health related quality of life survey are also promising. As a tool, the HRQOL survey provides an objective measure of the child and family/caregivers sense of self-efficacy and the external support services they are receiving. It can be used as a clinical tool to assess an individual family over time, as a tool for continuous quality improvement for the program, and as an approach to looking at the status of the community support system for families. The positive trend in this evaluation related to parental perceptions of quality of life that would be expected to be related to the length of enrollment in the Program validates, in part, the survey instrument, as does the lack of improvement in those areas that would not expected to be influenced by the length of enrollment in the Program, e.g., progression of disease. Several questions will need to be revised to ensure they are developmentally appropriate for the age of the child, e.g., awareness of disease progression. The higher scores for questions related to parent/caregiver self-efficacy, as compared to those related to external health system factors, provide insight as to the need and focus for future quality improvement endeavors.

Much was learned about the feasibility and limitations of several approaches to the research methods used in this initial study.

- The small sample size was a major factor in limiting conclusions. The impact assessment will be substantially enhanced if the evaluation is expanded to include all children enrolled in the PedsCare program in the future, and if it is extended to include multiple sites.
- Comparison of hospital utilization and cost pre- and post-enrollment in the Program is a viable approach to determining impact, though the disease course will negatively impact cost savings, as presumably over time the child's clinical status will worsen. Any positive impact on these types of variables will be somewhat moderated by the disease course. Even a modestly positive impact should therefore be interpreted as a significant gain.
- ICD 9 codes alone may not be adequate for identifying an appropriate comparison group for children enrolled in the PedsCare program, as the ICD 9 codes may not adequately

address severity of illness, a major factor in service utilization. Presumably the most ill children within an ICD 9 coding group will be in palliative care, so comparing children in and not-enrolled in a palliative care program may not result in the comparison of comparable groups. An additional approach to clarifying severity of illness is necessary to identify a comparison group to assess impact.

- Dose effect (the amount of time after enrollment required to produce an effect) may need to be determined before impact can be fully assessed. Future impact assessments could involve data collection with the HRQOL prior to client enrollment and at a standardized time after enrollment to assess the impact of the program.
- The HRQOL survey was developed using multiple "experts" and families. In general parents/caregivers scored high on questions. Revisions will need to be made over time with feedback from families and professionals to improve its sensitivity and validate the instrument.

Though the sample size was small, this initial study to evaluate the impact of community-based pediatric palliative care on hospital utilization and costs, and health related quality of life shows promising results. Future studies will require larger sample sizes, a longitudinal approach, consideration of length of enrollment on outcomes, validation of the HRQOL survey, and other methodological refinements. The importance of these future studies reflects the importance of the emerging role of pediatric palliative care to the health and well-being of children and the discipline of Pediatrics. In order to advance the practice of pediatric palliative and hospice care, it is imperative that further research generates an evidence base related to how to provide optimal services, and also how to train current practitioners and future health professionals.

This evaluation and report focus only on hospital utilization and costs and the health related quality of life of the family, two of the major areas of concern for the evaluation of palliative care programs. Other potential variables, e.g., the impact of the program on the number of hospitalizations over time; and emergency department, outpatient, subspecialty, etc. utilization and costs were not assessed. These variables represent important areas for future research.

REFERENCES

1. Bennini, F., et al., *Palliative care in children: problems and considerations.* Italian Journal of Pediatrics, 2004. 30: p. 205-209.
2. Carrol, J.M., et al., *Partners in Pediatric Palliative Care: A program to Enhance Collaboration between hospital and community palliative care services.* American Journal of Hospice and Palliaitve Medicine, 2007. 24(3): p. 191-195.
3. Contro, N., et al., *Family Perspectives on the Quality of Pediatric Palliative Care.* Archived Pediatric Adolescent Medicine, 2002. 156: p. 14-19.
4. Contro, N.A., et al., *Hospital Staff and Family Perspectives Regarding Quality of Pediatric Palliative Care.* Pediatrics, 2004. 114(5): p. 1248-1252.
5. Crawley, L.M., *Racial, Cultural, and Ethnic Factors Influencing End-of-Life Care.* JOURNAL OF PALLIATIVE MEDICINE, 2005. 8(Supplement 1).
6. Davis, M., et al., *The business of palliative medicine Part 2: The economics of acute palliative medicine.* American Journal of Hospice & Palliative Care, 2002. 19(2).
7. Dever, G.E.A., *Improving Outcomes in Public Health Practice: Strategy and Methods.* 1997, Gaithersburg: Aspen. 386.
8. Eiser, C. and R. Morse, *A review of measures of quality of life for children with chronic illness.* Arch Dis Child, 2001. 84: p. 205-211.
9. Francoeur, R.B., *A Flexible Item to Screen for Depression in Inner-City Minorities During Palliative Care Symptom Assessment.* American Journal Geriatric Psychiatry, 2006. 14: p. 228–236.
10. Goldman, A., *An Overview of Paediatric Palliative Care.* Medical Principles and Practice, 2007. 16(suppl 1): p. 46-47.
11. Gunten, C.F.v., et al., *Recommendations to improve end-of-life care through regulatory change in U.S. health care financing.* Journal of Palliative Medicine, 2002. 5(1): p. 35-41.
12. Hays, R.M., et al., *The Seattle Pediatric Palliative Care Project: Effects on Family Satisfaction and Health-Related Quality of Life.* Journal of Paliative Medicine, 2006.

9(3).

13. Himelstein, B.P., *Palliative Care for Infants, Children, Adolescents, and Their Families.* Journal of Palliative Medicine, 2006. 9(1).
14. Himelstein, B.P., et al., *Medical Progress Pediatric Palliative Care.* The New England Journal of Medicine, 2004. 350(17): p. 1752-1762.
15. Hinds, P.S., et al., *Key Factors Affecting Dying Children and Their Families.* JOURNAL OF PALLIATIVE MEDICINE, 2005. 8(Supplement 1): p. S-71-S77.
16. Hynson, J. and S. Sawyer, *Paediatric palliative care: Distinctive needs and emerging issues.* Journal of Paediatric Child Health, 2001. 37: p. 323–325.
17. Meier, D.E. and L. Beresford, *Pediatric Palliative Care Offers Opportunities for Collaboration.* JOURNAL OF PALLIATIVE MEDICINE, 2007. 10(2).
18. Seymour, J., et al., *Specialist palliative care: patients' experiences.* Journal of Advanced Nursing, 2003. 44(1): p. 24-33.
19. Sourkes, B., et al., *Food, Toys, and Love: Pediatric Palliative Care.* Current Problems of Pediatric Adolescent Health Care, 2005. 35(9): p. 350-386.
20. Tulsky, J.A., *Interventions to Enhance Communication among Patients, Providers, and Families.* JOURNAL OF PALLIATIVE MEDICINE, 2005. 8(Supplement 1): p. S-95-S102.

Appendix A

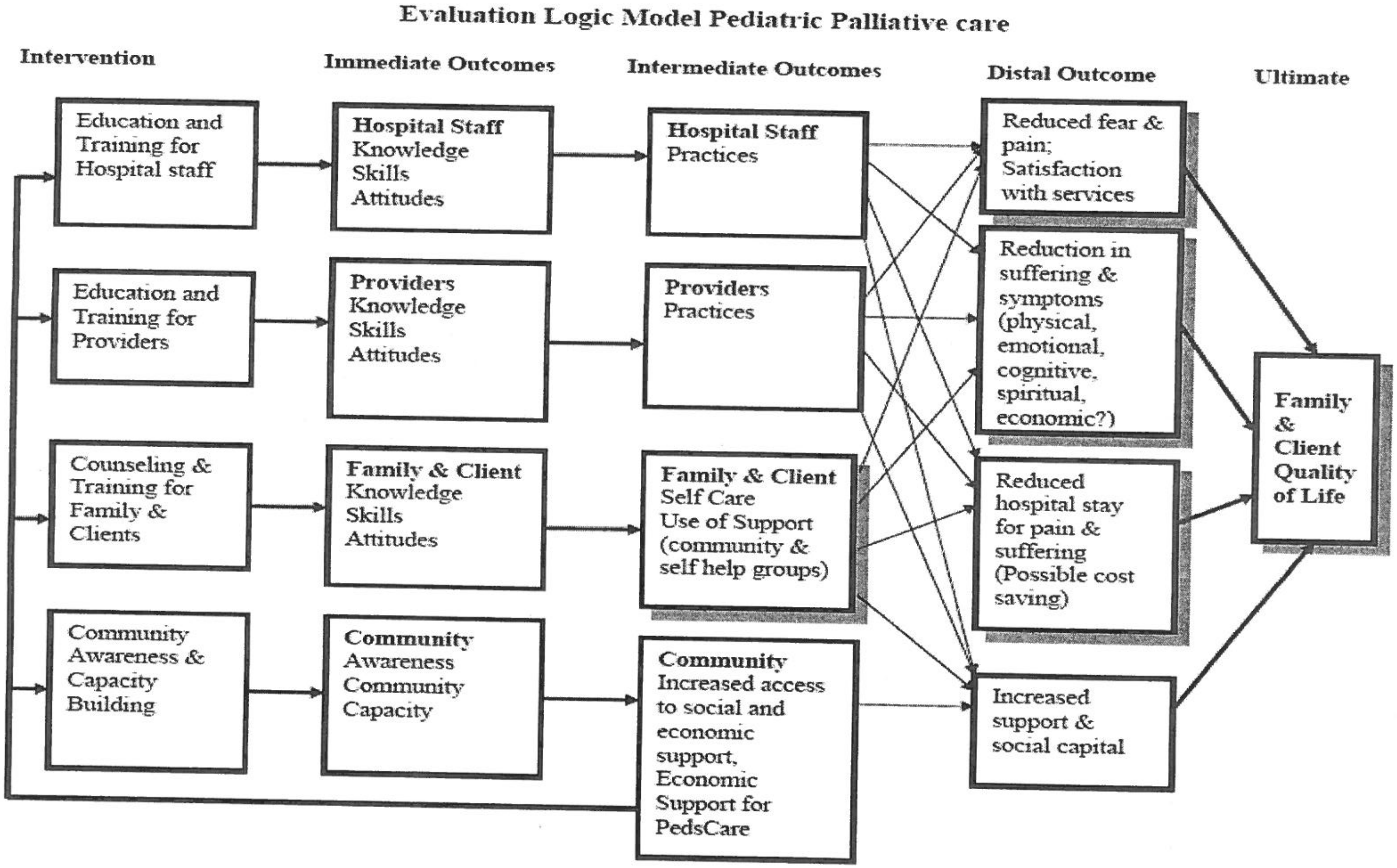

Evaluation Logic Model Pediatric Palliative care
Intervention
Immediate Outcomes
Intermediate Outcomes
Distal Outcome
Ultimate
Education and Training for Hospital staff
Education and Training for Providers
Counseling & Training for Family & Clients
Community Awareness & Capacity Building
Hospital Staff Knowledge Skills Attitudes
Providers Knowledge Skills Attitudes
Family & Client Knowledge Skills Attitudes
Community Awareness Community Capacity
Hospital Staff Practices
Providers Practices
Family & Client Self Care Use of Support (community & self help groups)
Community Increased access to social and economic support, Economic Support for PedsCare
Reduced fear & pain; Satisfaction with services
Reduction in suffering & symptoms (physical, emotional, cognitive, spiritual, economic?)
Reduced hospital stay for pain & suffering (Possible cost saving)
Increased support & social capital
Family & Client Quality of Life

APPENDIX B

Health Related Quality of Life Survey Results

Part I.

Community PedsCare Health Related Quality of Life	Day Range					
**General Emotional Health** How many days during the past 30 days have you felt...	**0 to 5 days**	**6 to 10 days**	**11 to 15 days**	**16 to 20 days**	**21 to 25 days**	**26 to 30 days**
1. ...stressed about your child's health	18 (34%)	11 (20.8%)	5 (9.4%)	2 (3.8%)	0 (0%)	17 (32.1%)
2. ...scared about your child's health	37 (69.8%)	6 (11.3%)	1 (1.9%)	0 (0%)	0 (0%)	9 (17%)
3. ...sad about your child's health	25 (47.2%)	8 (15.1%)	3 (5.7%)	2 (3.8%)	0 (0%)	15 (28.3%)
4. ...angry about your child's health	45 (84.9%)	3 (5.7%)	2 (3.8%)	0 (0%)	0 (0%)	3 (5.7%)
5. ...disappointed with my results when	46 (86.8%)	3 (5.7%)	2 (3.8%)	0 (0%)	0 (0%)	2 (3.8%)
**Respite Care** How many days in the last 30 days...						
6. Was there someone to relieve you of your role of taking care of your child?	25 (47.2%)	7 (13.2%)	2 (3.8%)	1 (1.9%)	0 (0%)	18 (34.0%)
**Activity Limitation** How many days during the past 30 days						
7. Were you not able to do your usual activities because of stress, depression, and other emotional problems	42 (79.2%)	4 (7.5%)	4 (7.5%)	0 (0%)	0 (0%)	3 (5.7%)

Part II.

Community PedsCare Health Related Quality of Life	None of the time	A little of the time	Sometimes	Most of the time	All the time
**Decision making**					
5. I am able to make good decisions concerning healthcare options for my child	0 (0%)	0 (0%)	1 (1.9%)	13 (24.5%)	39 (73.6%)
6. I am able to find a way to make sure that my child has healthcare specific to their needs	0 (0%)	0 (0%)	5 (9.4%)	13 (24.5%	35 (66.0%

Community PedsCare Health Related Quality of Life	None of the time	A little of the time	Sometimes	Most of the time	All the time
7. I receive correct information about my child's condition or illness	0 (0%)	1 (1.9%)	10 (18.9%)	11 (20.8%)	31 (58.5%)
8. I feel confident in my decision to manage my child's health	0 (0%)	0 (0%)	3 (5.7%)	14 (26.4%)	36 (67.9%)
9. I am satisfied with decisions made for my child's healthcare needs after a doctor's visit	0 (0%)	1 (1.9%)	8 (15.1%)	19 (35.8%)	25 (47.2%)
**Social Support**					
10. I have someone I can talk to about my fears concerning my child's health	1 (1.9%)	2 (3.8%)	7 (13.2%)	5 (9.4%)	38 (71.7%)
**Interaction/Communication**					
11. I can explain my child's need to my primary healthcare provider	0 (0%)	2 (3.8%)	4 (7.5%)	11 (20.8%)	36 (67.9%)
12. I can understand the needs of my child from my primary healthcare provider	0 (0%)	0 (0%)	4 (7.5%)	16 (30.2%)	33 (62.3%)
13. I am able to ask questions I may have about my child's healthcare	0 (0%)	0 (0%)	3 (5.7%)	7 (13.2%)	43 (81.1%)
14. My child has someone they can express themselves to when they are sad, angry, afraid, etc…	9 (17.0%)	0 (0%)	3 (5.7%)	4 (7.5%)	37 (69.8%)
**Access to Resources**					
I am able to obtain or have assistance in obtaining the following:					
15. ..Medicine	1 (1.9%)	0 (0%)	2 (3.8%)	6 (11.3%)	44 (83.0%)
16. ..Medical equipment	0 (0%)	0 (0%)	6 (11.3%)	13 (24.5%)	34 (64.2%)
17. ..Housing and Utilities	4 (7.5%)	0 (0%)	3 (5.7%)	4 (7.5%)	42 (79.2%)
**Child Health**					
18. I am able to understand the needs of my child	0 (0%)	0 (0%)	3 (5.7%)	16 (30.2%)	34 (64.2%)
19. My child understands their condition	29 (54.7%)	2 (3.8%)	7 (13.2%)	3 (5.7%)	12 (22.6%)
20. My child spends quality time with family and friends	1 (1.9%)	0 (0%)	4 (7.5%)	5 (9.4%)	43 (81.1%)
21. My child is treated with dignity while receiving healthcare services	0 (0%)	0 (0%)	1 (1.9%)	4 (7.5%)	48 (90.6%)

APPENDIX C . BAPTIST DATA DICTIONARY

**Variable Names**	
MRN-Medical Record Number	
Dschrg Date-Discharge date	
Patient Name	
Admit Date	
Birth Date	
**Diagnosis**	
Primary DX	Primary Diagnosis Name
Dx2	2nd Diagnosis Code
Dx3	3rd Diagnosis Code
Dx4	4th Diagnosis Code
Dx5	5th Diagnosis Code
Dx6	6th Diagnosis Code
Dx7	7th Diagnosis Code
Dx8	8th Diagnosis Code
Dx9	9th Diagnosis Code
Dx10	10th Diagnosis Code
Dx11	11th Diagnosis Code
Dx12	12th Diagnosis Code
Dx13	13th Diagnosis Code
Dx14	14th Diagnosis Code
Dx15	15th Diagnosis Code
**Payment Method by Customer-$$**	
Auto	Auto Insurance
Commercial	Commercial Insurance
Medicaid	Medicaid Insurance
Medicare	Medicare Insurance
Other	Other Insurance
Other Government	Other Government Insurance
Self Pay	Self Pay from Customer
Work Comp	Worker's Compensation Insurance
LOS	Length of Stay
Primary Insurance	Primary Insurance
Social Security Number	Social Security Number
Total Discharge	Total Charges at Discharge

Revenue Charges by Type		
Room and Board	R&B	Room and Board
	Nursery	Nursery
	NICU	Newborn, or Neonatal, Intensive Care Unit
	ICU or CCU	Intensive Care Unit/ Critical Care Unit
	OR	Operational Room
	Special Rooms	Other Room and Board
	Recovery Room	
Medical Equipment and Supplies	MedSurg Supplies Devices	Medical Surgical Supplies Devices
	Durable Med Equipment	Durable Medical Equipment
Laboratory Testing	Lab Other	Other Lab
	Lab Chemistry	Chemistry Lab
	Lab Immunology	Immunology Lab
	Lab Hematology	Hematology Lab
	Lab Bac Micro	Bacterial Microbiology
	Lab Urology	Urology Lab
	LabPathology	Pathology
Radiology Diagnostic Testing	Rad DX	Radiology Diagnosis
	CAT scans	
	Ultrasound	Ultrasound
	PETscan	PET scan
	MRI MRA	
Drug Therapy	Rad Therap Chemo	Radiation Therapy Chemotherapy
	Nuc Med	Nuclear Medicine
	IV Therapy	IV therapy
Physical Therapy	PT	Physical Therapy
	OT	Occupational Therapy
	Speech Therapy	Speech Therapy
Subspecialty Institutional Departments	Audiology	Audiology
	Cardiology	Cardiology
Electro diagnostics	EKG ECG	Electrocardiogram (diagnostic Test)
	EEG	Electroencephalogram (diagnostic Test)
Cardio-Stress Test	Card Cath Lab	Cardiac Catheter Lab
	Card Stress Test	Cardiac Stress Test
Dialysis		Kidney Diagnostic Testing
GI Services		Gastrointestinal Services
SpecialChrgs		Special Charges
Other Dx Services		Other Diagnosis services
Increm Nursing		Increment Nursing
ClinicLab		Clinic Lab
Pharmacy		Pharmacy

APPENDIX D

Diagnosis Detail for Community PedsCare Patients Served

Diagnosis Group	ICD 9 Code	Diagnosis Description
Blood/Blood Forming Organs	284.90	Aplastic anemia, unspecified
Central Urinary System	585.00	Chronic kidney disease
	586.00	Renal failure, unspecified
Circulatory System	416.00	Chronic pulmonary heart disease
	425.40	Other primary cardiomyopathies
	428.00	Heart failure
	429.90	Heart disease, unspecified
	440.90	Generalized and unspecified atherosclerosis
Congential Anomalies	741.00	Spina bifida
	742.10	Microcephalus
	742.20	Reduction deformities of brain
	742.30	Congenital hydrocephalus
	745.11	Double outlet right ventricle
	745.20	Tetralogy of Fallot
	746.70	Hypoplastic left heart syndrome
	748.50	Agenesis, hypoplasia, and dysplasia of lung
	751.30	Hirschsprung's disease and other congenital functional disorders of colon
	755.80	Other specified anomalies of unspecified limb
	756.00	Other congenital musculoskeletal anomalies
	756.51	Osteogenesis imperfecta
	756.60	Anomalies of diaphragm
	756.81	Absence of muscle and tendon
	758.00	Chromosomal anomalies
	758.20	Edward's syndrome
	759.70	Multiple congenital anomalies, so described
	759.90	Congenital anomaly, unspecified
Digestive System	573.90	Unspecified disorder of liver
	576.10	Cholangitis
Endocrine, Nutritional, Metabolism	269.90	Unspecified nutritional deficiency
	277.00	Cystic fibrosis
	277.02	With pulmonary manifestations
	277.80	Other specified disorders of metabolism
	277.86	Peroxisomal disorders
	277.87	Disorders of mitochondrial metabolism
	279.40	Autoimmune disease, not elsewhere classified

Diagnosis Group	ICD 9 Code	Diagnosis Description
Injury	959.01	Head injury, unspecified
	994.10	Drowning and nonfatal submersion
	996.81	Kidney
	996.83	Heart
Muscular Skeletal System	728.88	Rhabdomyolysis
Neoplasms	170.00	Malignant neoplasm of bone and articular cartilage
	191.00	Malignant neoplasm of brain
	191.80	Other parts of brain
	191.90	Brain, unspecified
	192.20	Spinal cord
	194.40	Pineal gland
	195.10	Thorax
	199.10	Other, Cancer unspecified site
	202.30	Malignant histiocytosis
	205.00	Myeloid leukemia
	237.70	Neurofibromatosis
Nervous System/Sense Organs	330.00	Cerebral degenerations usually manifest in childhood
	330.10	Cerebral lipidoses
	335.10	Spinal muscular atrophy
	335.19	Other, Spinal muscular atrophy
	343.80	Other specified infantile cerebral palsy
	343.90	Infantile cerebral palsy, unspecified
	345.60	Infantile spasms
	348.10	Anoxic brain damage
	348.30	Encephalopathy, not elsewhere classified
	359.10	Hereditary progressive muscular dystrophy
Perinatal Period Conditions	765.00	Disorders relating to short gestation and low birth weight
	765.10	Other preterm infants
	770.70	Chronic respiratory disease arising in the perinatal period
	777.50	Necrotizing enterocolitis in fetus or newborn
Respiratory System	518.84	Acute and chronic respiratory failure
Symptoms, Signs, Ill Defined	783.40	Lack of expected normal physiological development in childhood
	783.41	Failure to gain weight
	794.31	Abnormal electrocardiogram [ECG] [EKG]

APPENDIX E

QUALITY IMPROVEMENT: PROCESS MEASURES

Existing processes were observed from archived data, PedsCare Provider Team meetings and Program Administration meetings. In an effort to expand and enhance existing processes, opportunities for quality improvement in Healthcare services and Professional Development for PedsCare Staff were identified. Categorical measurements were identified for 5 categories within Healthcare Services and for 1 category for Professional Development to serve as problem identification tools for the pediatric palliative care program (Table 8: Proposed Process Measures Indicators).

Table 8
Proposed Process Measure Indicators

Observation Type	Process Types	Design Measure	Diagram Display
Prospective Clients*	Healthcare Services	Frequency by Yearly Dates	Run Chart
Days between Referral and Admission		Quarterly Means	Control Chart
Education Sessions for Family Caregiver		Quarterly Means	Observation/ Surveys
Customer Satisfaction		Prevalence Scores	Bar Chart
Number of Assisted Doctor's Visits by PedsCare Provider Type		Quarterly Means	Run Chart
Literature Review Sessions	Professional Development	Quarterly Means	Pie or Bar Chart

*Referred only, enrollment pending
Prepared by Duval County Health Department: Institute for Health, Policy and Evaluation Research Health Care Services

Healthcare Services

During data collection, existing variables within Community Hospice's electronic medical records were identified as key monitoring variables for continuous quality improvement. These variables were: Prospective Clients (clients who were referred but have a pending enrollment status), Referral Date, Admission Date, and Primary Care Giver Names (Name of Parent/Guardian of the Pediatric special needs child). Counts of prospective clients are a potential tool to measure timely access into the PedsCare program. These measures were available through the PedsCare database (variable: Prospective Clients). A run chart is a potential problem analysis tool that could be used to display the counts of clients who had a pending admission status within a selected month of service (Figure 6).

Figure 6. Prospective Clients for Pediatric Palliative Care Services -September 2007

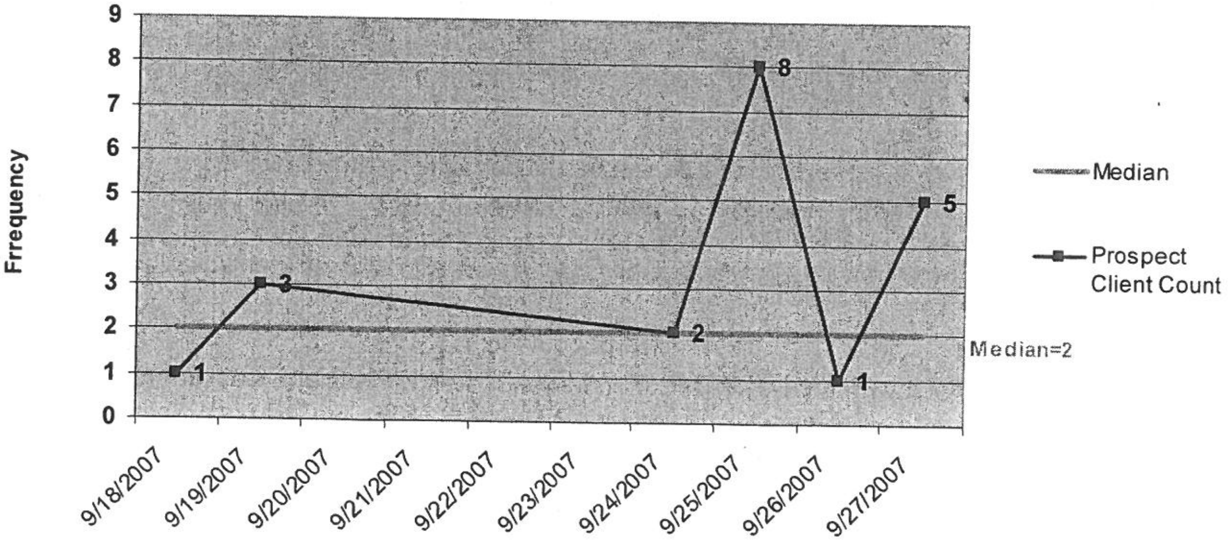

Days between referral and admission dates could also be calculated. A control chart could serve as a future problem analysis tool for the illustration of healthcare access to palliative care services (Figure 7).

Figure 7. Average Days Between Referrals and Admissions Into Pediatric Palliative Care By Year

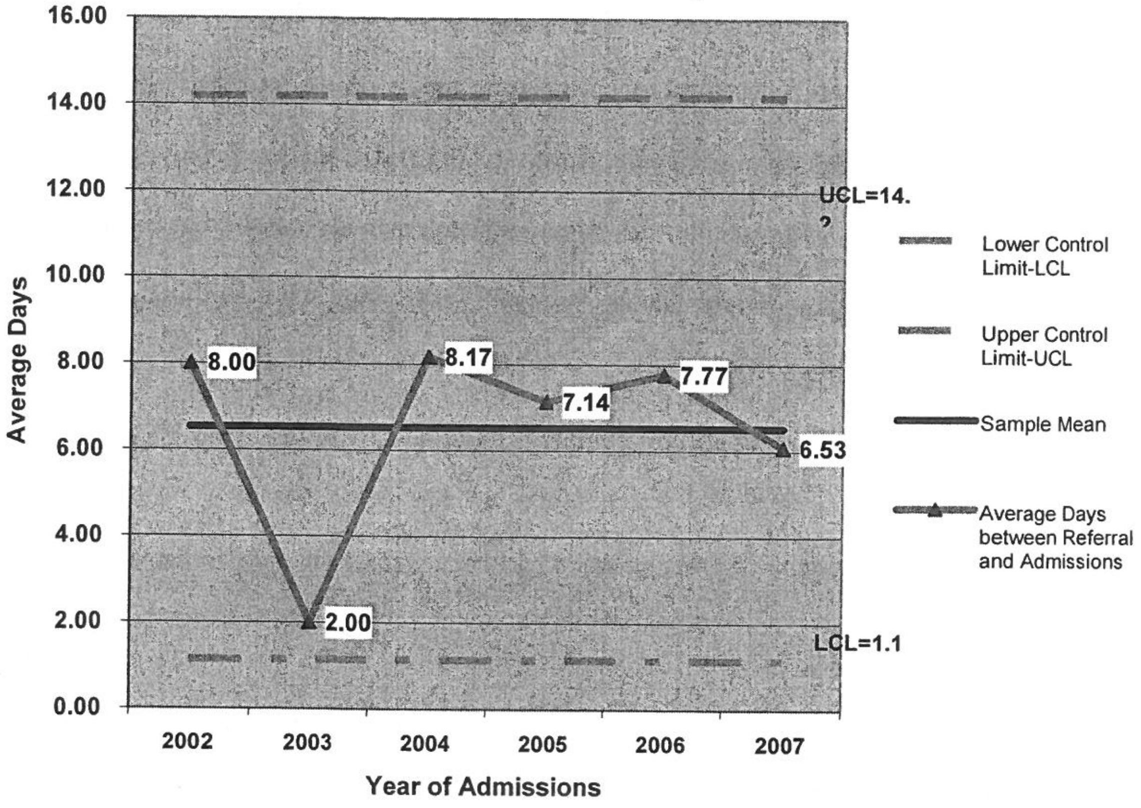

Key service variables [Number of Assisted Doctor's Visits and Educations Sessions for family caregiver(s)] were not located in the existing database. These variables could be added as drop fields within Community Hospice's electronic medical records database to later serve as problem identification tools. The illustration of quarterly averages of assisted doctors' visits by PedsCare provider type and education sessions for family caregivers could serve as a link to quality improvement of services to family care givers

(parents/guardians of pediatric clients enrolled in Palliative Care) to intermediate outcomes of self care and use of support (Appendix A- Logic Model) .

Furthermore, a customer satisfaction survey could be used as a quantifiable outcome measure to address customer needs and satisfaction with delivery of services. Responses from Family Caregivers could be collected by Community PedsCare Volunteers. The analysis from customers' responses could be used to link existing and new processes for palliative care coordination with the more ultimate goal of palliative care (families' and clients' quality of life) (Appendix A).

Professional Development

Scientific literature reviews could be used in addition to existing training for Community PedsCare staff monthly review of scientific literature reviews. The number of scientific articles presented and discussed among Community PedsCare team could serve as a way to quantify and illustrate averages of reviewed articles that could later be displayed monthly at team meetings (Table 9). These process measures will aid in the effort to link quality of services to the ultimate outcome of Palliative Care Services (Quality of life for family and client). Table 9 serves as an aid in data collection and/or analysis of existing and new processes of palliative care coordination.

Table 9

PedsCare Data Tools for Process Measures

Data Tools:	Collect	Display	Analysis
**Problem Identification Tools:**			
▪ Surveys (Written, Phone interview)	X	X	X
**Problem Identification/Problem Analysis Tool:**			
▪ Run Chart		X	X
▪ Pareto Chart		X	X
**Problem Analysis Tools:**			
▪ Pie and bar charts		X	X
▪ Control Charts (Attribute)		X	X

Prepared by Duval County Health Department: Institute for Health, Policy and Evaluation Research

Institutional Review Committee
Kathy Seabolt, Coordinator

BAPTIST HEALTH

March 21, 2008

The attached protocol will expire in approximately 60 days. Please complete your Continuing Review or Study Closure report and forward to the Baptist Medical Center Institutional Review Committee (Howard Bldg, Suite 413). The Application for Continuing Review or Study Closure Form is attached. If you are renewing your study, please forward a clean copy of your consent document (if applicable). Renewals cannot be presented to the board without this document.

Reports must be returned to the Institutional Review Committee Coordinator no later than 3 weeks prior to the next regularly scheduled committee meeting. Any renewals received after the meeting deadline will be held for the next regularly scheduled meeting. Protocols actively enrolling patients are not eligible for Expedited Review and must be presented to the full board.

Failure to respond by the protocol renewal date will result in suspension of all research activities until such a time as full board approval is obtained. Any exceptions will be discussed with the Chairman of the Institutional Review Committee at 202-2127. Protocols that have not been renewed and are more than 30 days past the expiration date will be closed by the IRB, necessitating resubmission as a new protocol.

If the protocol is more than 60 days past the expiration date, it will be considered continuing non-compliance and will be reported to the Office of Human Research Protections.

Your prompt response is greatly appreciated. Please feel free to contact me if you have any questions.

Sincerely,

Kathy Seabolt

Kathy Seabolt
Coordinator
Institutional Review Committee

Baptist Medical Center Institutional Review Committee

Application for Continuing Review or Study Closure

Please complete ALL sections of this form whether applying for Continuing Review or Study Closure

Sponsor Name / No.: **Baptist Health Systems Foundation / No.:**

Date of IRB Expiration: **6/24/2008**

Principal Investigator: **William Livengood, PhD**

Study Title: **The Impact of Pediatric Palliative Care Coordination on the Quality of Life of Patients with Life Limiting Conditions, The Impact of Pediatric Palliative Care.**

07-42

Renewal Status of the Researc

Select the one choice that best describes the current state of this research study:

- ☐ No participants have been enrolled to date.
- ☐ Recruitment and/or enrollment of new participants or review of records/specimens continue.
- ☐ Study is no longer enrolling, but participants still receive research related interventions, (e.g., still receiving treatment, obtaining blood draws)
- ☐ Study is no longer enrolling and participants have completed research related interventions. Study remains active only for long term follow-up.
- ☑ Study enrollment is permanantly closed, participants have completed all research related interventions and long term follow-up has been completed. The remaining research activities are limited only to data analysis that may require contact with records and specimens.

Close the Study

Please provide a final study report, progress reports, and publications to the IRB as they become available.

☑ Close the Study. Enrollment and follow-up are complete and no further contact with participants/records/specimens is anticipated

Summary of Progress since the previous IRB Continuing Review Approval *

58 Total number of participants consented to participate to date, including withdrawals

58 Total number of participants consented since the previous IRB continuing review approval *

5 Total number of participants consented but did not complete the study since the previous IRB continuing review approval * (include explanation for each)

Provide a description of study activities to date, including any difficulties in recruiting subjects, in the space below:

Recruited and consented participants through the phone. Not all participants could be recruited because the phone was disconnected (n=6) or was the wrong phone number (n=1). Five participan[illegible] missed scheduled phone interview and could not be contacted after multiple atten[illegible] of calling them to reschedule at another time.

Select one answer for each question:

1. ☐ Yes ☑ No — Is an independent Safety Monitor or Data Safety Monitoring Board (DSMB) assigned to periodically review data from this study for risks to participants?

If 'Yes' How often does the monitor or board perform review?

Please attach all data safety or progress reports completed since the previous IRB continuing review approval. *

2. ☐ Yes ☑ No — Since the previous IRC continuing review approval have any unanticipated problems involving risks to participants and /or serious unanticipated and research related adverse events occurred at sites where a BMC/WCH investigator is involved in the conduct of the research or is responsible for regulatory reporting?

Total number of events/problems since previous IRC continuing review N/A

☐ Yes ☐ No **Have these events/problems been reported previously to the IRC?**

* IRB initial study or continuing review approval

Baptist Medical Center Institutional Review Committee

Application for Continuing Review or Study Closure

Please complete ALL sections of this form whether applying for Continuing Review or Study Closure

Sponsor Name / No.: **Baptist Health Systems Foundation / No.:**

Date of IRB Expiration: **6/24/2008**

Principal Investigator: **William Livengood, PhD**

Study Title: **The Impact of Pediatric Palliative Care Coordination on the Quality of Life of Patients with Life Limiting Conditions, The Impact of Pediatric Palliative Care.**

07-42

3. ☑ Yes ☐ No — Is this a multi-center study? If 'No", skip to question # 4, if 'Yes' :

☐ Yes ☑ No — Since the previous IRB continuing review approval, have any unanticipated problems involving risks to participants and /or serious unanticipated and reserach related adverse events occurred at sites where a BMC/WCH investigator is not involved in the conduct of the research or is not responsible for regulatory reporting?

Total number of events/problems since previous IRC continuing review approval

If 'Yes attach all written summaries and/or progress reports completed since the previous IRB continuing review approval.

4. ☑ Yes ☐ No — Have any relevant clinical findings, literature, reports or other information (particularly information about risks associated with the research) become available since the last IRB review approval? If 'Yes' please describe:

A report was submitted to the DuPont foundation. Summarizing the assessment of family/caregivers perceptions of Health Related Quality of Life. Also, a cost and utilization study was conducted to assess hospital cost and utilizati

5. ☐ Yes ☑ No — Have there been any complaints about this research since the last IRB review approval? If 'Yes' please describe:

6. ☐ Yes ☑ No — Did the IRC require the use of a written informed consent document for this study?

Investigator's Conflict of Interest Statement

☐ Yes ☑ No — Do you or any other person responsible for the design, conduct or reporting of the research have an economic interest in, or act as an officer or a director of any outside entity whose financial interests would reasonably appear to be affected by the research?

If 'Yes' and the IRB has not yet been notified, submit a letter to the IRB describing the conflict immediately.

In addition to the above responses, I confirm that a current IRB-approved consent form has been signed, dated, and is retained in my files for every participant enrolled in this study and a copy was provided to the person who signed the form (if use of a consent form was required). I also confirm that no changes to study procedures or the consent form(s) were initiated without prior IRB approval.

[signature]

Principal Investigator's Signature

4/ /08

Date

William Livengood, PhD
Phone #: (904) 222-1682

* IRB initial study or continuing review approval

BAPTIST
Medical Center Downtown

820 Prudential Drive, Suite 413
Jacksonville, Florida 32207
Phone: 904.202.2127
Fax: 904.202.2331
www.e-baptisthealth.com

Institutional Review Committee

April 22, 2008

William Livengood, PhD
Institute for Health, Policy & Evaluation Research
Duval County Health Department
900 University Boulevard North
Suite 604
Jacksonville, FL 32211

RE: **#07-42, Baptist Health Foundation, "The Impact of Pediatric Palliative Care Coordination on the Quality of Life of Patients with Life Limiting Conditions: The Impact of Pediatric Palliative Care".**

Dear Dr. Livengood:

The following was reviewed and approved via expedited review per 45 CFR 46.110 by the Baptist Medical Center (BMC) Institutional Review Committee (IRC) Chairman on April 21, 2008:

-Close the study. Enrollment and follow-up are complete and no further contact with participants/records/specimens is anticipated.

This study will be removed from our active files per your request. If you have any questions, please contact the IRC office immediately. The BMC IRC meets the requirements in 21 CFR 56 (Rev.), 45 CFR 46 (Rev.) and ICH (E6) GCP guidelines.

Sincerely,

Michael Joyce, MD, PhD
Chairman
Institutional Review Committee

BAPTIST
Medical Center Downtown

820 Prudential Drive, Suite 413
Jacksonville, Florida 32207
Phone: 904.202.2127
Fax: 904.202.2331
e-baptisthealth.com

Institutional Review Committee

RECEIVED
AUG - 9 2007

August 8, 2007

William Livingood, Ph.D.
Duval County Health Department
900 University Boulevard North
Suite 604
Jacksonville, FL 32211

Dear Dr. Livingood:

The Institutional Review Committee (IRC) of Baptist Medical Center (BMC) met on August 8, 2007, and the following new protocol was reviewed and approved via expedited review for a period of one year:

> **#07-42, Baptist Health Foundation, "The Impact of Pediatric Palliative Care Coordination on the Quality of Life of Patients with Life Limiting Conditions: The Impact of Pediatric Palliative Care".**
> -HIPAA Consent Document, no version date.

The anniversary date for this study will be August 7, 2008. At that time, please submit a report of your experiences with this protocol. This protocol qualifies for a waiver of obtaining informed consent, per 45 CFR 46.117(c)(2).

Should you have any questions, please contact the IRC office. The BMC IRC meets the requirements in 21 CFR 56 (Rev.), 45 CFR 46 (Rev.) and ICH (E6) GCP guidelines.

Good luck with this endeavor.

Sincerely,

Michael J. Joyce

Michael Joyce, MD, PhD
Chairman
Institutional Review Committee

Kimberly -
The letter I sent yesterday had the wrong date on it. Can you replace it with this corrected letter?
Thanks,
Kathy

FLORIDA DEPARTMENT OF
HEALTH

Charlie Crist
Governor

Ana M. Viamonte Ros, M.D., M.P.H.
Secretary of Health

INSTITUTIONAL REVIEW BOARD NON-RESEARCH DETERMINATION

July 19, 2007

William C. Livingood, PhD
Director, Institute for Health, Policy and Evaluation Research
Duval County Health Department
900 University Blvd Suite 604 MC 99
Jacksonville, FL 32211

Protocol Title: An Outcome Evaluation of Pediatric Palliative Care: Cost and Utilization Study
Protocol Number: H07171

IRB Determination: Activity does NOT involve human subject's research

Based on the information provided, the Department of Health Institutional Review Board, or representative, determined your activity does not involve research, as defined in DOH policy and federal regulation, to mean "systematic investigation...designed to develop or contribute to generalizable knowledge" (§ 45 CFR 46.102(d)).

As a reminder, if there is a change in the activity, IRB review may become necessary. If you have questions about whether your activity may require IRB approval, please contact the IRB administrative office so we may determine whether the additional activities come under the category of research.

If you have any questions, or if we can be of any assistance, please contact the Department of Health IRB at (850) 245-4585 or toll-free in Florida (866)-433-2775. You may also visit our website at: http://www.doh.state.fl.us/execstaff/irb/

Thank you for your cooperation with the IRB.

Sincerely,

Robert Hood

Robert Hood, Ph.D.
Assistant Director
Office of Public Health Research

Federal Wide Assurance#: 00004682

UF | Institutional Review Board
UNIVERSITY of FLORIDA

Health Science Center / Jacksonville
College of Medicine
Institutional Review Board

580 West Eighth Street
Tower II, 9th Floor, Suite 9015
Jacksonville, Fl 32209
(904) 244-9427
(fax) (904) 244-9035

FWA00005790

MEMORANDUM

DATE: July 25, 2007

TO: William Livingood, Jr., PhD
600 University Blvd., Ste 604-MC-99
Jacksonville,, FL 32211

FROM: Sheila Heim, CIP
Coordinator, Institutional Review Board for
Alan Halperin, MD
Chair, Institutional Review Board

SUBJECT: Expedited Review of UFJ 2007-76

TITLE: UFJ 2007 076 The Impact of Pediatric Palliative Care Coordination on the Quality of Life of Patients with Life Limiting Conditions

Your request for approval of the above study under the classification of expedited has been reviewed and as IRB Chair I am pleased to inform you that your study is now approved under the expedited category(s):

___1. Clinical studies of drugs and devices only when:
a. An investigational new drug application (IND) or investigational device exemption (IDE) is not required, and there is no significant increase in risk or decrease in acceptability of risk, or
b. The device is cleared or approved for marketing and is being used in accordance with its labeling.

___ 2. Collection of blood samples by finger, heel, or ear stick, or venipuncture no more than twice weekly as follows:
a. From healthy non-pregnant adults weighing at least 110 pounds, in amounts less than 550 ml per 8 weeks.
b. From other adults and children, considering the health and habitus of the subjects, in amounts less than 50 ml or 3 ml per kg (whichever is less) per 8 weeks.

___ 3. Prospective collection of biological specimens for research purposes by noninvasive means. Examples:
a. Hair and nail clippings (non-disfiguring).
b. Deciduous teeth at exfoliation or indicated extraction
c. Permanent teeth excreta at indicated extraction
d. Excreta and external secretions including sweat
e. Uncannulated saliva
f. Placenta removed at delivery
g. Amniotic fluid at the time of rupture of the membrane prior to or during labor
h. Supra- and sub-gingival dental plaque during routine prophylactic scaling
i. Mucosal and skin cells by buccal scraping or swab, skin swab, or mouth washings.
j. Sputum after saline mist nebulization

An Equal Opportunity Institution

___ 4. Collection of data through noninvasive procedures (not involving general anesthesia or sedation) routinely employed in clinical practice, excluding procedures involving x-rays or microwaves. Examples:
a. Physical sensors that do no involve input of significant amounts of energy or invasion of privacy.
b. Weighing or testing sensory acuity.
c. Electro-cardiography, electro-encephalography, thermography, detection of naturally- occurring radioactivity, electroretinography, ultrasound, diagnostic infrared imaging, doppler blood flow, and echocardiography.
d. Moderate exercise, muscular strength testing, body composition assessment and flexibility testing where appropriate for age, weight and health.

_✓_ 5. Research involving materials (data, documents, records, specimens) that have been or will be collected solely for non-research purposes (such as medical treatment or diagnosis).

___ 6. Collection of data from voice, video, digital, or image recordings made for research purposes.

___ 7. Research on individual or group characteristics or behavior (such as studies of perception, cognition, motivation, identity, language, communication, cultural beliefs and practices, and social behavior) or research employing survey, interview, oral history, focus group, program evaluation, human factors evaluation, or quality assurance methodologies.

You must inform the Board of any modifications or changes to this research (protocol or consent changes) since they could affect its expedited status.

Please note the category of informed consent listed below that has been approved for this study.

_____ You have been granted approval to conduct this study using the enclosed stamped, IRB-approved consent form. This consent must be photocopied and used when enrolling subjects into this project.

OR

_____ You have been granted a waiver of documentation of informed consent, in lieu of a verbal consent.

OR

__✓__ You have been granted a waiver of informed consent.

Your protocol is approved until 7/27/2008 at which time you will need to submit a regular continuing review report in order to continue the study.

Thank you for informing the Board of your proposal.

An Equal Opportunity Institution

BAPTIST
Medical Center Downtown

820 Prudential Drive, Suite 413
Jacksonville, Florida 32207
Phone: 904.202.2127
Fax: 904.202.2331
e-baptisthealth.com

Institutional Review Committee

August 8, 2007

William Livingood, Ph.D.
Duval County Health Department
900 University Boulevard North
Suite 604
Jacksonville, FL 32211

Dear Dr. Livingood:

The Institutional Review Committee (IRC) of Baptist Medical Center (BMC) met on June 25, 2007, and the following new protocol was reviewed and approved via expedited review for a period of one year:

> **#07-42, Baptist Health Foundation, "The Impact of Pediatric Palliative Care Coordination on the Quality of Life of Patients with Life Limiting Conditions: The Impact of Pediatric Palliative Care".**
> -HIPAA Consent Document, no version date.

The anniversary date for this study will be August 7, 2008. At that time, please submit a report of your experiences with this protocol. This protocol qualifies for a waiver of obtaining informed consent, per 45 CFR 46.117(c)(2).

Should you have any questions, please contact the IRC office. The BMC IRC meets the requirements in 21 CFR 56 (Rev.), 45 CFR 46 (Rev.) and ICH (E6) GCP guidelines.

Good luck with this endeavor.

Sincerely,

Michael Joyce, MD, PhD
Chairman
Institutional Review Committee

IRB UFJ
Palliative Care

FLORIDA DEPARTMENT OF
HEALTH

Charlie Crist
Governor

Ana M. Viamonte Ros, M.D., M.P.H.
State Surgeon General

INSTITUTIONAL REVIEW BOARD NON-RESEARCH DETERMINATION

October 8, 2007

William C. Livingood, Ph.D.
Institute for Health, Policy and Evaluation Research
Duval County Health Department
900 University Blvd., Suite 604 MC 99
Jacksonville, FL 32211

Protocol Title: The Outcome Evaluation of Pediatric Palliative Care: Health Related Quality of Life

IRB Decision: Program Evaluation - Activity does NOT involve research

Based on the information provided, the Department of Health Institutional Review Board, or representative, determined your activity does not involve research, as defined in DOH policy and federal regulation, to mean "systematic investigation…designed to develop or contribute to generalizable knowledge" (§ 45 CFR 46.102(d)).
As a reminder, the decision regarding classification lies in the intent of the project. If the primary intent is to generate generalizable knowledge, the project is research. If the primary intent is to prevent or control disease or injury or to improve a public health program, and no research is intended at the present time, the project is non-research. If the primary intent changes to generating generalizable knowledge, then the project becomes research, and it is important that the Investigator submit a proposal to the IRB for review and approval prior to release of such information. If you have questions about whether your activity may require IRB approval, please contact the IRB administrative office so we may determine whether the additional activities come under the category of research.
If you have any questions, or if we can be of any assistance, please contact the Department of Health IRB at (850) 245-4585 or toll-free in Florida (866)-433-2775. You may also visit our website at: http://www.doh.state.fl.us/execstaff/irb/

Thank you for your cooperation with the IRB.

Sincerely,

Robert Hood

Robert Hood, Ph.D.
Assistant Director
Office of Public Health Research

Federal Wide Assurance#: 00004682

109

University of Florida Health Science Center/Jacksonville • Institutional Review Board

INTRODUCTORY QUESTIONNAIRE – EXEMPT STUDIES

Please provide contact information for a representative who can answer any questions that the IRB might have concerning this submission:	
Name:	Radley Remo
Position:	Research Associate
E-mail:	Radley_remo@doh.state.fl.us
Phone #:	904-253-2052
Pager #:	
**2nd Contact**:	Susie Coughlin 904-253-2054

This box is for IRB use ONLY.

IRB Project #: ______________

This form is ONLY for specific types of studies as described below under Type of Project. Review that information carefully before completing this application.

TYPE OF PROJECT

Check the type of project below and complete ALL items (1-11) on this form. The Exempt Project section should only be used if you are applying for Exempt Review.

☒ **Exempt**
- Under "Exempt Project" (last section on this form), check the applicable criterion.
- Attach a copy of any data collection form(s) or questionnaire(s) that will be used.

☐ **Tissue or Data Bank**
- See "Use and Storage of Specimens and/or Data from Human Subjects for Current or Future Research" posted at http://rgp.ufl.edu/irb/irb01/.
- A "Confidentiality Agreement for Specimens" must be used when the specimens are used by other investigators.

☐ **Training Grant.** Attach a copy of the grant.

☐ **Administrative or Statistical Center Project.** Attach a copy of pertinent administrative policies and procedures for the Center.

☐ **Non-Human Subject Research**
- "Human subject" is defined as a living individual about whom a researcher obtains (1) data through intervention or interaction with the individual or (2) identifiable private information.
- Non-human subject research means there is (1) NO intervention or interaction with a living person, including both physical procedures and manipulation of the subject or subjects' environment, that are performed for research purposes, and (2) NO identifiable private data or information obtained in a form associable with the individual. "Associable" means that the identity of the individual is or may readily be ascertained or associated with the information obtained from that individual
- Contracts or sub-contracts for data analysis. If data are/will be collected at a non-UF site and will be sent to you for analysis, provide a copy of the IRB approval letter, Protocol, and Informed Consent Form from the site where data are being obtained.

PROJECT TITLE: Access to Care for Medicaid Clients in Duval County *

****Note**: If there is grant support for this project, the grant title and the IRB project must have the exact same title.*

Date(s) of deadline(s) for grants or funding related to this project: [ ]

INVESTIGATOR INFORMATION

PRINCIPAL INVESTIGATOR (PI) INFORMATION

NAME. LAST: Livingood **FIRST:** William **MIDDLE:** C

DEGREE: MD ☐ DMD/DDS ☐ JD ☐ PhD ☒ MS/MA ☐ BS/BA ☐ Other ☐ None ☐

POSITION: Faculty ☒ Staff ☐ Student ☐

Other; Describe: [ ]

COLLEGE: University of Florida

DEPARTMENT: Pediatrics

CAMPUS ADDRESS: [ ]

PHONE: 904-253-2339

FAX: 904-665-3111

EMAIL: william_livingood@doh.state.fl.us

BEEPER: [ ]

DEPARTMENT/THESIS CHAIR: [ ]

SUB-INVESTIGATOR(S). List as: Last name, First Name/Initial, Middle Name/Initial, Degree.

Remo, Radley C., MPH, Coughlin, Susan, MPH

CONTACT PERSON IF NOT THE PRINCIPAL INVESTIGATOR

NAME, DEGREE, AND POSITION: Radley C. Remo, MPH Research Associate

PHONE: 904-253-2052

FAX: 904-665-3111

EMAIL: radley_remo@doh.state.fl.us

BEEPER: [ ]

Check here if there has been a change in any of the above information since your last IRB submission: ☒

Principal Investigator Assurance

I certify that the information provided in this application is complete and correct.

As Principal Investigator, I have the ultimate responsibility for conducting the study and doing so ethically, for protecting the rights and welfare of the human subjects, and for strictly adhering to any stipulations imposed by the Institutional Review Board.

I agree to comply with all UF policies and procedures as well as all applicable federal, state, and local laws regarding the protection of human subjects in research, including, but not limited to the following:

- Ensuring that only qualified personnel conduct the study according to the approved Protocol
- Implementing no changes in the approved Protocol or Informed Consent Form without prior Institutional Review Board approval, except in an emergency, if necessary to safeguard the well-being of human subjects.
- Obtaining legally effective informed consent from human subjects or their legally responsible representative and using only the currently approved, stamped Informed Consent Form, when applicable.
- Promptly reporting serious and unexpected adverse events to the Institutional Review Board in writing within 5 working days of occurrence or notification of occurrence.
- Completing investigator training as required by the Institutional Review Board.
- If I will be unavailable to conduct or direct this research personally, as when on sabbatical, leave, vacation or termination of employment, arranging for a co-investigator to assume direct responsibility in my absence. The Institutional Review Board will be notified in writing of this change through a formal revision in the IRB project that is submitted ***in advance***.

William C. [illegible] 7/25/2007

Principal Investigator Signature Date

Chair Person or Director of Department Signature

I approve this project for submission to the Institutional Review Board.

Chairperson or Director of Department Signature Date

STUDY PROCEDURES

A. PROTOCOL INFORMATION

1. Explain the purpose of the study: The purpose of this study is to analyze data from a surveillance effort to monitor the impact of Medicaid Reform on access to health care providers and compare the results to similar results from other communities. The percent of physicians who are on the Medicaid provider list and are currently accepting new Medicaid patients is the outcome measure. Comparisons between results from primary care and specialty physicians will be conducted.
2. Describe the research procedures. Be ***very*** explicit and complete: The researchers will analyze previously collected data on Medicaid providers. A number of statistical test such as chi square and t-tests will be used to analyze the data.
3. By what authority does the Principal Investigator and sub-investigator(s) have access to the medical record, specimen, or subject? The researchers have access to secondary data that have been reported and will further analyze.

B. HUMAN SUBJECT INVOLVEMENT: No ☒ Yes ☐. ***If yes,*** complete the rest of this section.

4. Type of subjects or subject information to be studied, for example: healthy volunteers, patients with specific diseases, medical records, or pathological specimens: ☐
5. If medical records or pathological specimens are to be used, please specifically indicate the dates involved. For example: "We wish to review medical records/pathological specimens from January 1998 through December 2002": ☐
6. Number of subjects/medical records/specimens. This number should (a) be based on requirements for statistical power if possible and (b) allow for loss of data through, for example, screening failures: ☐
7. Gender of subjects
   a. The following will be enrolled: Males ☐ Females ☐ Both ☐
   b. If only one gender is to be included, check one of the following:
      ☐ Only the gender selected has the condition.
      ☐ Other, please specify: ☐

8. Race/ethnicity

a. Will subjects of a specific race/ethnicity be recruited? No ☐ Yes ☐. ***If Yes, specify:***

b. Type: Caucasian ☐ African-American ☐ Hispanic ☐ Asian ☐ Other ☐

c. Reason (check one):

☐ The condition being studied occurs only in the selected group(s).

☐ Other, please specify: ☐

9. Will vulnerable subjects be considered for participation in this study? No ☐ Yes ☐

If Yes, specify: ☐ Prisoners

☐ Pregnant Women/Fetuses

☐ Children

☐ Impaired subjects

☐ Other: ☐

10. Investigator-subject relationship. Will the PI be the subjects':

a. Physician? No ☐ Yes ☐ NA ☐

b. Instructor or advisor? No ☐ Yes ☐ NA ☐

c. Supervisor? No ☐ Yes ☐ NA ☐

C. Procedures for Protecting Confidentiality and Privacy

11. Procedures for collecting and recording data.

a. What identifying information will be collected and written on your data collection form?

No data will be collected. The researcher will analyze secondary data. The dataset will be housed on a separate secure password protected server. This server will have limited access. To protect privacy, the dataset will be stripped of personal identifiers. All names will be numerically coded and saved to a different file. The code sheet will then be saved on a separate password protected folder.

b. Will you use a coding system to protect subjects' identities? No ☐ Yes ☒. ***If Yes:***

Will there be a key to the code and what type of code will it be? Yes, a numeric code.

c. Will it be possible for you or any study staff to go back and identify a subject after the data collection has been completed? No ☐ Yes ☐

12. Will this study use specimens, records, or data that are linked to the subject's identity, including a coded list? No ☒ Yes ☐.

If Yes, please explain: ☐

a. List the sources of any individually identifiable specimens, records, or data. Please be ***very*** specific: ☐

b. Do all of these materials exist, now, at the time of this submission, or will some be obtained from subjects in the future? (NOTE: in order for a study to qualify for exemption, the materials must be in existence PRIOR to the beginning of the project) ☐

c. What procedures will you use to obtain the materials? ☐

d. Please include a copy of data collection form(s) and/or questionnaire(s):

☐ NA

☐ Data collection form(s) attached

☐ Questionnaire(s) attached

☐ Other form(s) attached. Please list: ☐

D. Conflict of Interest

13. Do you, the University of Florida, or any of the sub-investigators hold a patent or license for any material, object, or process used in this project? ☐ Yes. ☒ No.

14. Is a patent or license pending or under consideration or is there any intention to file a patent application at a later date? ☐ Yes. ☒ No.

15. Do you, the University of Florida, or any of the sub-investigators own stock in the company sponsoring the project? ☐ Yes. ☒ No.

16. Do you or any of the sub-investigators give presentations for or serve as a consultant to the sponsoring company on their behalf? ☐ Yes. ☒ No.

17. Do you or any of the sub-investigators have any other possible conflict of interest? ☐ Yes. ☒ No.

    If yes, explain: ☐

E. HIPAA

18. Request for IRB/Privacy Board Waiver of HIPAA authorization requirement.

    a. If you checked yes in number 12 above, you *must* complete the following (i-v) to request IRB/Privacy Board Waiver of HIPAA authorization. If you checked "no" in number 12 above, you *may* complete the following (i-v) to request waiver of HIPAA authorization *or you may* proceed to 18b and provide a statement from a statistician certifying that the data is sufficiently anonymized/de-identified to satisfy IRB and HIPAA requirements. **Note:** For your protocol to proceed you **must** receive either a waiver of authorization under 18a *or* provide a statement from a statistician certifying that the data is sufficiently anonymized/de-identified under 18b.:

        (1) If you are requesting a Waiver of the Informed Consent or a Waiver of Documentation of Informed Consent you must complete the following items (I-VI):

            I. The following protected health information will be created, collected, used or disclosed as a result of the subject's participation in this research (see note to PIs below):

               ☐

Investigators: Please delete this box before submitting!

NOTE TO PI:
Investigator must list/describe the protected health information that will be created, used and/or disclosed to determine the subject's eligibility for the study and information obtained through the course of the study. The list/description must contain sufficient specificity to identify the information in a specific and meaningful fashion. The following list provides examples and categories for investigators to consider listing here (Note, as a general rule, the more sensitive the information, the more specifically it should be described, i.e. HIV results):

- Complete past medical history to determine eligibility criteria listed in informed consent
- Information about HIV/AIDS
- Information about hepatitis infection
- Information about sexually transmitted diseases
- Information about other infectious diseases that must be reported to Public Health authorities
- Records of physical exams
- Laboratory, x-ray, MRI, and other test results
- Diaries and questionnaires
- Records about study medications or drugs
- Records about study devices
- Information related to diagnosis and treatment of a mental health condition

II. The use or disclosure of protected health information involves no more than a minimal risk to the privacy of individuals based on, at least, the following elements:

A. Is there an adequate plan to protect subject identifiers from improper use and disclosure?
☐ No
☐ Yes – please describe: ☐

B. Is there an adequate plan to destroy the identifiers at the earliest opportunity consistent with the conduct of the research, unless there is a health or research justification for retaining the identifiers or such retention is otherwise required by law?
☐ No
☐ Yes – please describe: ☐

C. Protected Health Information (PHI) will not be reused or disclosed to any person or entity, except as required by law, for authorized oversight of the research study, or for other research for which use or disclosure of PHI would per permitted under HIPAA regulations.

☐ Agree

☐ Disagree

III. The research could not practicably be conducted without the waiver or alteration.

☐ Agree

☐ Disagree

IV. The research could not practicably be conducted without access to and use of PHI.

☐ Agree

☐ Disagree

V. Use and disclosure of PHI under this waiver is necessary until (insert date here).

b. If you checked "no" in number 12 above, and do not complete section 18, you must provide a statement from a statistician certifying that the data is sufficiently anonymized/de-identified to satisfy IRB and HIPAA requirements. Thus, it is important for investigators to remove from their data collection any subject identifiers (including age, gender, etc.) that are not *absolutely necessary* for their research.

EXEMPT PROJECT

Please indicate under which criterion you are requesting exempt review for this protocol. ***Important note:* *For pediatric subjects***, criterion 2 only applies when educational tests are being studied or when public behavior is being observed and the investigator does not participate in the activities being observed.

☐ 1. Research conducted in established or commonly accepted educational settings, involving normal educational practices, such as:
(i) Research on regular and special education instructional strategies, or
(ii) Research on the effectiveness of or the comparison among instructional techniques, curricula, or classroom management methods.

☐ 2. Research involving the use of educational tests (cognitive, diagnostic, aptitude, achievement), survey or interview procedures, or the observation of public behavior, so long as confidentiality is maintained. If ***both*** of the following are true, exempt status cannot be granted:

(i) Information obtained is recorded in such a manner that the subject can be identified, directly or through identifiers linked to the subject, **and**

(ii) Subject's responses, if known outside the research could reasonably place the subject at risk of criminal or civil liability or be damaging to the subject's financial standing or employability or reputation. (*An example of this would be research that deals with sensitive aspects of the subject's own behavior that could be illegal or socially unacceptable, such as drug use, sexual behavior, or use of alcohol, or that the subject might not want publicly known, for any reason.)*

☐ 3. Research involving the use of educational tests (cognitive, diagnostic, aptitude, achievement), survey procedures, interview procedures, or observation of public behavior that is not exempt under #2, if:

(i) The human subjects are elected or appointed public officials or candidates for public office; or

(ii) Federal statute(s) require(s) without exception that the confidentiality of the personally identifiable information will be maintained throughout the research and thereafter.

☒ 4. Research involving the collection or study of ***existing*** data, documents, records, pathological specimens or diagnostic specimens, if these sources are publicly available or if the information is recorded by the investigator in such a manner that subjects cannot be identified, directly or through identifiers linked to the subjects. ***"Existing"*** means already collected and/or stored before your study starts, not that collection will occur as part of routine care.

☐ 5. Research and demonstration projects, which are conducted by or subject to the approval of department or agency heads, and which are designed to study, evaluate, or otherwise examine:

(i) Public benefit or service programs;

(ii) Procedures for obtaining benefits or services under those programs;

(iii) Possible changes in or alternatives to those programs or procedures; or

(iv) Possible changes in methods or levels of payment for benefits or services under those programs.

☐ 6. Taste and food quality evaluation and consumer acceptance studies, if:

i. Wholesome foods without additives are consumed, OR

ii. A food is consumed that contains a food ingredient at or below the level and for a use found to be safe, or agricultural chemical or environmental contaminant at or below the level found to be safe, by the Food and Drug Administration, or approved by the Environmental Protection Agency, or the Food Safety and Inspection Service of the U.S. Department of Agriculture.

☐ NOT RESEARCH

************************END OF APPLICATION***********************

Institutional Review Board
UNIVERSITY *of* FLORIDA

Health Science Center / Jacksonville
College of Medicine
Institutional Review Board

580 West Eighth Street
Tower II, 9th Floor, Suite 9015
Jacksonville, Fl 32209
(904) 244-9427
(fax) (904) 244-9035

FWA00005790

MEMORANDUM

DATE: October 1, 2007

TO: William Livingood, Jr., PhD
900 UNIVERSITY BLVD. STE 604-MC-99
Jacksonville,, FL 32211

FROM: Sheila Heim, CIP SAH
Coordinator, Institutional Review Board for
Alan Halperin, MD
Chair, Institutional Review Board

SUBJECT: Expedited Review of UFJ 2007-132

TITLE: UFJ 2007 0132 An outcome evaluation of pallative care: Health related quality of life

Your request for approval of the above study under the classification of expedited has been reviewed and as IRB Chair I am pleased to inform you that your study is now approved under the expedited category(s):

___1. Clinical studies of drugs and devices only when:
a. An investigational new drug application (IND) or investigational device exemption (IDE) is not required, and there is no significant increase in risk or decrease in acceptability of risk, or
b. The device is cleared or approved for marketing and is being used in accordance with its labeling.

___ 2. Collection of blood samples by finger, heel, or ear stick, or venipuncture no more than twice weekly as follows:
a. From healthy non-pregnant adults weighing at least 110 pounds, in amounts less than 550 ml per 8 weeks.
b. From other adults and children, considering the health and habitus of the subjects, in amounts less than 50 ml or 3 ml per kg (whichever is less) per 8 weeks.

___ 3. Prospective collection of biological specimens for research purposes by noninvasive means. Examples:
a. Hair and nail clippings (non-disfiguring).
b. Deciduous teeth at exfoliation or indicated extraction
c. Permanent teeth excreta at indicated extraction
d. Excreta and external secretions including sweat
e. Uncannulated saliva
f. Placenta removed at delivery
g. Amniotic fluid at the time of rupture of the membrane prior to or during labor
h. Supra- and sub-gingival dental plaque during routine prophylactic scaling
i. Mucosal and skin cells by buccal scraping or swab, skin swab, or mouth washings.
j. Sputum after saline mist nebulization

An Equal Opportunity Institution

___ 4. Collection of data through noninvasive procedures (not involving general anesthesia or sedation) routinely employed in clinical practice, excluding procedures involving x-rays or microwaves. Examples:
a. Physical sensors that do no involve input of significant amounts of energy or invasion of privacy.
b. Weighing or testing sensory acuity.
c. Electro-cardiography, electro-encephalography, thermography, detection of naturally- occurring radioactivity, electroretinography, ultrasound, diagnostic infrared imaging, doppler blood flow, and echocardiography.
d. Moderate exercise, muscular strength testing, body composition assessment and flexibility testing where appropriate for age, weight and health.

___ 5. Research involving materials (data, documents, records, specimens) that have been or will be collected solely for non-research purposes (such as medical treatment or diagnosis).

___ 6. Collection of data from voice, video, digital, or image recordings made for research purposes.

_✓_ 7. Research on individual or group characteristics or behavior (such as studies of perception, cognition, motivation, identity, language, communication, cultural beliefs and practices, and social behavior) or research employing survey, interview, oral history, focus group, program evaluation, human factors evaluation, or quality assurance methodologies.

You must inform the Board of any modifications or changes to this research (protocol or consent changes) since they could affect its expedited status.

Please note the category of informed consent listed below that has been approved for this study.

_____ You have been granted approval to conduct this study using the enclosed stamped, IRB-approved consent form. This consent must be photocopied and used when enrolling subjects into this project.

OR

_____ You have been granted a waiver of documentation of informed consent, in lieu of a verbal consent.

OR

__✓__ You have been granted a waiver of informed consent.

Your protocol is approved until 10/1/2008 at which time you will need to submit a regular continuing review report in order to continue the study.

Thank you for informing the Board of your proposal.

An Equal Opportunity Institution
